# Inverted strand polarity yields thermodynamically stable G-quadruplexes and prevents duplex formation within extended DNA†

**DOI:** 10.1039/d3sc05432b

**Published:** 2024-08-27

**Authors:** Bruce Chilton, Ruby J. Roach, Patrick J. B. Edwards, Geoffrey B. Jameson, Tracy K. Hale, Vyacheslav V. Filichev

**Affiliations:** a School of Food Technology and Natural Sciences, Massey University Private Bag 11-222 Palmerston North 4442 New Zealand v.filichev@massey.ac.nz

## Abstract

DNA G-quadruplexes (G4) formed in guanine-rich sequences play a key role in genome function and maintenance, interacting with multiple proteins. However, structural and functional studies of G4s within duplex DNA have been challenging because of the transient nature of G4s and thermodynamic preference of G-rich DNA to form duplexes with their complementary strand rather than G4s. To overcome these challenges, we have incorporated native nucleotides in G-rich sequences using commercially available inverted 3′-O-DMT-5′-O-phosphoramidites of native nucleosides, to give 3′-3′ and 5′-5′ linkages in the centre of the G-tract. Using circular dichroism and ^1^H nuclear magnetic resonance spectroscopies and native gel electrophoresis, we demonstrate that these polarity-inverted DNA sequences containing four telomeric repeats form G4s of parallel topology with one lateral or diagonal loop across the face of the quadruplex and two propeller loops across the edges of the quadruplex. These G4s were stable even in the presence of complementary C-rich DNA. As an example, G4 assemblies of inverted polarity were shown to bind to the hinge region of Heterochromatin Protein 1α (HP1α), a known G4-interacting domain. As such, internal polarity inversions in DNA provide a useful tool to control G4 topology while also disrupting the formation of other secondary structures, particularly the canonical duplex.

## Abstract 2

Telomeric DNA strands with inverted sequence polarity form stable G-quadruplexes (G4s) even in the presence of complementary DNA and interact with G4-binding domain of Heterochromatin Protein 1α.

## Graphical abstract

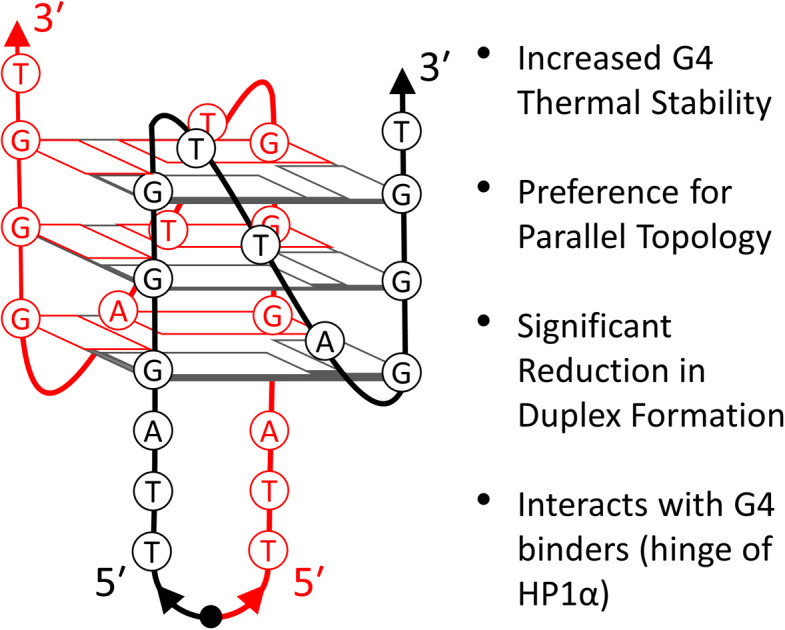

## 1. Introduction

G-quadruplexes (G4s) are non-canonical secondary structures of DNA that form in guanine (G)-rich regions of genomic DNA with a general consensus sequence of (G_*n*_X)_4_, where X can be any combination of nucleotides and *n* is two or more. ChIP-G4 seq showed that within chromatin there are over 10 000 potential G4-forming regions, which are enriched in gene promoters and transcriptional regulation.^1,2^ Not surprisingly, many transcription factors and chromatin binding proteins, such as Heterochromatin Protein 1α (HP1α),^3^ have been shown to interact with G4s, reinforcing the importance of understanding their role in genome function.

**Fig. 1:**
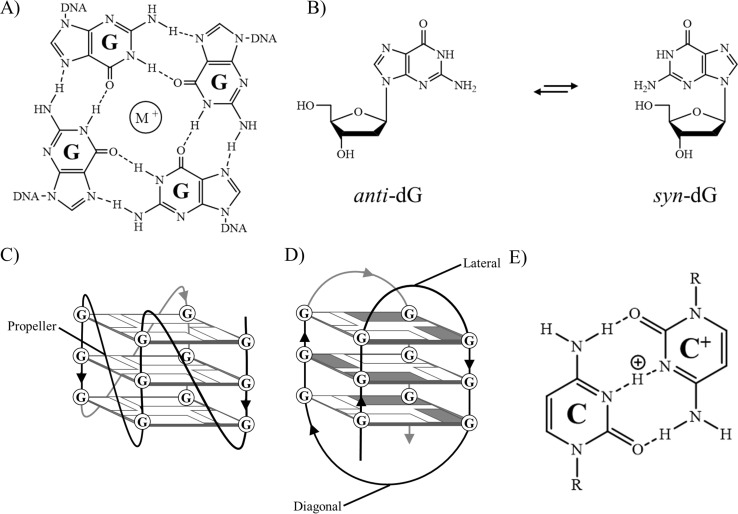
(A) Hydrogen-bonding arrangement of guanosine nucleobases to form a G-tetrad unit in G-quadruplexes. (B) *anti*- and *syn*-configurations of guanosine's glycosidic torsion angle. (C) A G-quadruplex of parallel topology containing propeller loops only. (D) A G-quadruplex of antiparallel topology containing lateral and diagonal loops; guanine bases as rectangles; guanines with *anti*-glycosidic angle are white; guanines with *syn*-glycosidic angle are grey. (E) Hydrogen-bonding arrangement of cytosine and protonated cytosine to form a C:C^+^ base pair in an i-motif. R represents the ribose sugar and phosphate, connecting to the larger DNA sequence.

The G4 structure is composed of stacked arrangements of quartets of guanines, referred to as G-tetrads. A G-tetrad is a square arrangement of four hydrogen-bonded guanines arranged around a central cation, typically Na^+^ or K^+^ (Fig. 1A).^4,5^ Two or more of these tetrads can stack on top of each other, forming G4s which are highly polymorphic, with different orientations of strands and loops depending on the loop sequences, pH, salt concentration or level of molecular crowding. Parallel G4s feature G-tracts all oriented in the same direction and have purely *anti*-guanosine glycosidic linkages, whereas antiparallel topologies have G-tracts oriented in opposite directions and a mixture of *syn*- and *anti*-guanosine glycosidic bonds (Fig. 1B–D). Additionally, structures may differ in the length or orientation of loops, such as lateral, diagonal or propeller loops.

When duplex DNA is unwound, while the G-rich strand can form a G4, the complementary C-rich strand also has the potential to form a secondary structure known as an i-motif.^6^ This structure is typically detected at a slightly acidic pH (approximately 5.5) as cytosine has to be hemi-protonated, forming C:C^+^ base pairs (Fig. 1E). i-Motifs can also be formed at neutral pH.^7^

Both G4 and i-motif structures have been detected *in vivo*.^8^ Immunostaining human MCF-7 breast cancer cells showed that G4s were predominantly observed during the S phase and i-motifs were observed during G1 phase. Induced formation of one structure using ligands known to stabilise that secondary structure reduced formation of the other, suggesting that formation was mutually exclusive. *In vitro*, single-molecule force-ramp assays, using optical tweezers, were carried out on DNA containing complementary G- and C-rich regions on opposing strands.^9^ Structures were stretched and the forces required to unfold the secondary structures for sequences containing complementary G- and C-rich strands were compared in different buffers expected to allow formation of different structures. When both structures were expected to form, the force-extension curves indicated that only one secondary structure was ever present. However, when the two structures were offset sufficiently, rather than being directly opposite each other, both structures were observed. The prevalence of i-motif forming regions in the genome suggests that these structures could also have an important role in cellular function.^6,7^

G4s and i-motifs are recognised by many proteins found in cells, although the role of interactions of these structures with cellular components is not well understood from structural and functional perspectives. G4-interacting proteins that are necessary for genome function and maintenance include protein HP1α that maintains heterochromatin,^3^ DNA methyltransferases, such as DNMT3A,^10^ a DNA helicase Pif1,^11^ and many more.^12^*In vitro* biophysical experiments have shown that these interactions are topology specific.^3,13^

Further studies of these interactions require the ability to trap G4 topologies within duplex DNA strands, creating a more accurate model of DNA in cell nuclei. Studying non-canonical structures in these contexts, including large nucleosomal arrays, will advance our understanding of their importance in the genome. However, the free energy (Δ*G*) values for G4 formation compared to duplex formation primarily indicate that under physiological pH, temperature, and salt concentrations the canonical duplex is favoured over G4 structures.^14–16^ Single-molecule FRET experiments indicate that in solutions containing G-rich sequences and complementary C-rich sequences, a mixture of secondary structures is observed.^17^ Molecular crowding has been reported to encourage G4 formation,^18^ potentially indicating some preference for G4 structures in packed cellular environments. This suggests that G4 formation is transient in nature and obtaining accurate data requires the creation of large, accurate models of genomic DNA containing thermodynamically stable G4s. Inducing and controlling G4 and i-motif formation *in vitro* and *in vivo* is therefore a subject of considerable research. Several small-molecule ligands have been shown to stabilise certain non-canonical secondary structures, including G4s,^19^ but these ligands could interfere with protein-binding sites. These factors have to be considered when studying structural aspects of G4–protein interactions. Modified nucleotides, such as 2′-fluororibonucleic acid, have also been considered to create kinetically trapped G4s,^20^ but over time and upon heating and cooling down in the presence of the complementary strand, DNA duplexes are formed as the thermodynamic product.^21^

**Fig. 2:**
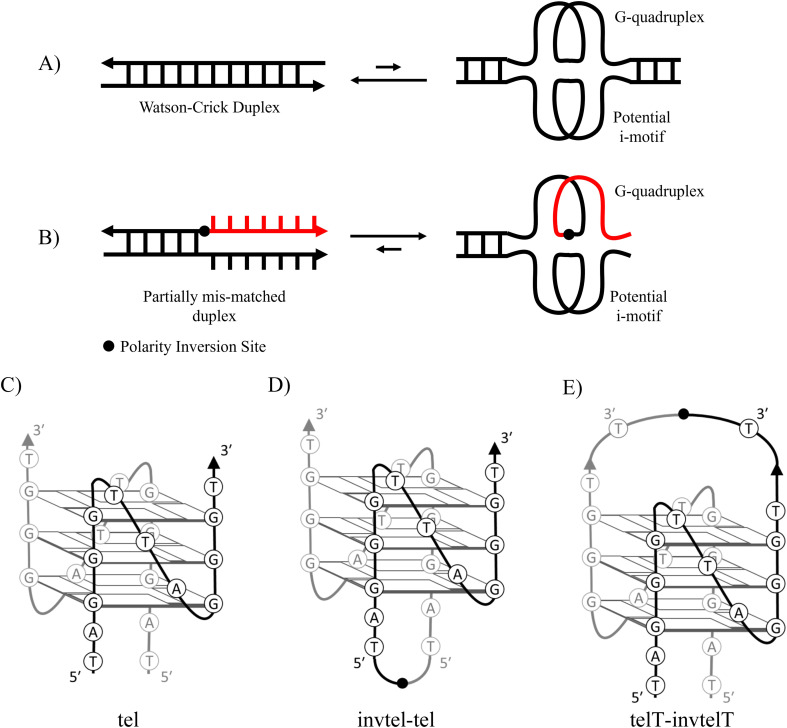
(A) Equilibrium established between native duplex and a G-quadruplex, favouring the formation of the canonical duplex. (B) Proposed arrangement of DNA containing polarity inversions, potentially favouring the formation of a G-quadruplex, native nucleotides are shown in black, the polarity-inverting 3′-3′ or 5′-5′ nucleotide links are shown in red. (C) Parallel bimolecular telomeric G4 (tel) characterised by NMR spectroscopy. (D) A proposed structure of a monomeric telomeric sequence containing an inversion at the 5′-end (invtel-tel). (E) A proposed structure of a monomeric telomeric sequence containing an inversion at the 3′-end (telT-invtelT).

We hypothesised that DNA duplexes containing a folded thermodynamically stable G4 in the presence of a complementary C-rich strand could be obtained by introducing a polarity inversion in the G-rich sequence, 5′-3′-3′-5′ or 3′-5′-5′-3′. Such sequences create mismatches in a canonical duplex without affecting G4 formation (Fig. 2A and B). Polarity inversion can be accomplished using commercially available 3′-O-DMT-5′-O-phosphoramidites of native nucleosides, where the canonical positions of the DMT protecting group at 5′ and the phosphoramidite linking group at 3′ are interchanged. These modifications would not affect glycosidic bond configurations and G-tract orientations compared to native DNA. The only differences are the interaction with complementary DNA and the presence of a diagonal loop, which is absent in simple 5′-3′ DNA sequences predisposed to form parallel G4s. Canonical duplexes are antiparallel meaning the strands are oriented in opposite directions. With polarity inversion, some nucleotides will always be in an unfavourable orientation for duplex formation.

We have demonstrated that these inversions are not only able to stabilise G4s and affect their topology, they also almost completely disrupt duplex formation. However, the effective change to the topology/loop arrangements of the modified G4 has a negative impact on the binding of the HP1α protein used to test the interactions of modified G4s with a known G4-binding protein.

## 2. Results and discussion

### 2.1. Structural considerations for design of G4s with inverted polarity

Previously, the effect of polarity inversions on G4 stability and topology had been investigated in the context of tetramolecular G4s^22,23^ formed by TG_*n*_T (*n* = 3–5) and monomeric thrombin-binding aptamer.^23,24^ This involved inversion of one or two nucleotides in the sequence which did not affect the original G4 topology according to circular dichroism (CD) and nuclear magnetic resonance (NMR) spectra. We used a 12-mer telomeric repeat that forms a bimolecular G4 primarily adopting a parallel topology in K^+^ buffers according to NMR data (Fig. 2C).^25,26^ The symmetrical nature of this assembly makes it an ideal G4 to study as it simplifies NMR spectra. The two sequences run in opposite directions from the 5′-end and visual analysis of the characterised structure indicates that the terminal 5′-dT nucleotides are close to each other in space. We proposed to link them with a native phosphate and create a G4 with a polarity inversion containing a diagonal loop (Fig. 2D, sequence invtel-tel, Table 1). We expected that the original G4 structure would be maintained while reinforcing the parallel topology and reducing formation of the canonical duplex in the presence of the complementary strand (Fig. 2B). To account for any increase in steric hindrance caused by the introduction of this additional loop, we also designed an elongated sequence to allow for extra flexibility in the loop (sequence invTtel-Ttel, Table 1). In addition, we tested sequences which contained an inversion at the 3′-end instead of the 5′-end (Fig. 2E), with both one- and two-nucleotide extensions since the 3′-ends of the native sequence were slightly further apart than the 5′-ends (sequences telT-invtelT and telTA-invtelTA, respectively, Table 1). The unmodified sequences, forming bimolecular G4s, were used as controls in our study. We refer to the non-inverted telomeric sequence, as shown in Table 1, as tel and the sequences with inverted polarity according to the location of their inversions (*i.e.* invtel-tel or telT-invtelT). Elongated sequences are referenced by the location and nucleotide(s) used for elongation (*e.g.* Ttel is the tel sequence with an additional thymidine at each 5′-end, whereas telT refers to the tel sequence having extra T at each 3′-end). We also anticipated that the increased strand length and formation of uni- rather than bimolecular G4s would increase the thermal stability of resulting complexes. To ensure that the observed effects were the result of polarity inversion, we used a negative control sequence, called letT-Ttel, which mimicked the nucleotide composition of invTtel-Ttel. However, in the absence of polarity inversions one of the two Ttel sequences is changed from 5′-3′ to 3′-5′, consequently forming an antiparallel G4 topology, according to CD. DNA sequences complementary to G4s are also provided in Table 1. Their names include prefix “c-” to denote C-rich sequences.

**Table 1:** Native and inverted G4-forming sequences based on human telomeric repeat

Name	Sequence	Complementary sequence, 5′-3′	Name of complementary sequences^a^
tel	5′-(TAG_3_T)_2_-3′	(AC_3_TA)_2_	c-tel
Ttel	5′-T(TAG_3_T)_2_-3′	(AC_3_TA)_2_A	c-Ttel
telT	5′-(TAG_3_T)_2_T-3′	A(AC_3_TA)_2_	c-telT
telTA	5′-(TAG_3_T)_2_TA-3′	TA(AC_3_TA)_2_	c-telTA
invtel-tel	3′-(TG_3_AT)_2_-5′—5′-(TAG_3_T)_2_-3′	(AC_3_TA)_2_(ATC_3_A)_2_	c-invtel-tel
invTtel-Ttel	3′-(TG_3_AT)_2_T-5′—5′-T(TAG_3_T)_2_-3′	(AC_3_TA)_2_AA(ATC_3_A)_2_	c-invTtel-Ttel
telT-invtelT	5′-(TAG_3_T)_2_T-3′—3′-T(TG_3_AT)_2_-5′	(ATC_3_A)_2_AA(AC_3_TA)_2_	c-telT-invtelT
telTA-invtelTA	5′-(TAG_3_T)_2_TA-3′—3′-AT(TG_3_AT)_2_-5′	(ATC_3_A)_2_ATTA(AC_3_TA)_2_	c-telTA-invtelTA
letT-Ttel	5′-(TG_3_AT)_2_TT(TAG_3_T)_2_-3′	(AC_3_TA)_2_AA(ATC_3_A)_2_	c-invTtel-Ttel

a There is no polarity inversion for the C-rich complementary sequences to the G-rich sequences with polarity inversion.

### 2.2. Polarity inversions in telomeric G4s induce higher thermal stability

We investigated the influence of polarity inversion on the G4 topology, molecularity, and thermal stability using CD spectroscopy and non-denaturing PAGE. The composition and purity were confirmed by mass spectrometry (Fig. SI-1, Table SI-1†) and denaturing PAGE (dPAGE, 7 M urea, Fig. SI-2†). G4s give characteristic CD profiles depending on their topology, resulting from the arrangement of guanosines having *syn-* and *anti*-conformations of the glycosidic bond. The presence of only *anti*-guanosines results in strong peaks at 265 nm and 240 nm with positive and negative ellipticity, respectively, which is a characteristic profile of a parallel G4. Antiparallel G4s contain a mixture of glycosidic bond configurations, indicated by positive peaks at 290 and 240 nm and a negative peak at 265 nm in CD spectra.^27–30^ Telomeric G4 sequences tend to favour antiparallel topologies in Na^+^-containing buffers, and parallel topology when K^+^ ions are added.

**Fig. 3:**
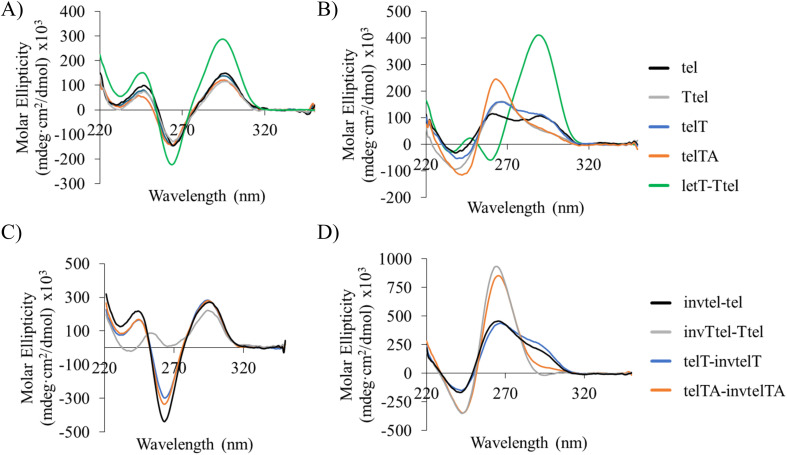
CD profiles of native and modified tel sequences at room temperature: (A) unmodified sequences in Na^+^ buffer; (B) unmodified sequences in K^+^ buffer; (C) polarity-inverted sequences in Na^+^ buffer; (D) polarity-inverted sequences in K^+^ buffer. Here and elsewhere, Na^+^ buffer is 20 mM sodium phosphate, pH 7.0; K^+^ buffer is 20 mM sodium phosphate, 10 mM KCl, pH 7.0; strand concentration: 20 μM.

Unmodified G4s showed the characteristic peaks of antiparallel G4s in Na^+^ buffer (Fig. 3A), but in K^+^ buffer they formed a mixture of parallel and antiparallel topologies with a positive peak at 265 nm, a negative peak at 240 nm and a positive shoulder or a peak at 290 nm (Fig. 3B). DNA melting data shown in Table 2 also showed distinct *T*_1/2_ values for individual structures. A control sequence letT-Ttel had, as expected, the signature of antiparallel G4 in both buffers, a significant difference to the native bimolecular G4s.

**Table 2:** *T*_1/2_ values of native and inverted G4-forming sequences based on human telomeric repeat in the presence of Na^+^ or K^+^ ions^a^

Sequence	*T*_1/2_ (Na^+^, °C, ±3 °C)	*T*_1/2_ (K^+^, °C, ±3 °C)
tel	37 (ap)	37 (ap), 48 (p)
Ttel	34 (ap)	48 (p)
telT	38 (ap)	40 (ap), 45 (p)
telTA	38 (ap)	50 (p)
invtel-tel	76 (ap)	83 (p)^(i)^
invTtel-Ttel	34 (ap), 75 (p)	84 (p)^(i)^
telT-invtelT	68 (ap)	74 (p)
telTA-invtelTA	62 (ap)	73 (p)
letT-Ttel	51 (ap)	58 (ap)

a G4 topology: (p) denotes parallel topology, (ap) denotes antiparallel topology. (i) Estimated value as complex was only partially melted at 90 °C.

In Na^+^ buffer the sequences containing polarity inversions gave antiparallel CD profiles similar to the parent unmodified sequences, except for invTtel-Ttel, which gave a small positive peak at 255 nm (Fig. 3C). The overall shape, position and relative intensities of CD peaks of invTtel-Ttel are similar to those in the CD spectrum of G_3_T_4_G_3_ sequence, which forms a bimolecular antiparallel G4 with one G-quartet having the opposite hydrogen bond polarities to the other two Q-quartets (PDB structure 1FQP).^31,32^ However, as with the unmodified sequences, the addition of K^+^ ions resulted in a significant shift towards the parallel topology. The invtel-tel and telT-invtelT sequences gave mixed topologies similar to the unmodified parent sequences, but invTtel-Ttel and telTA-invtelTA with lengthened diagonal loops shifted completely to a parallel topology in K^+^ buffer, lacking the shoulder at 290 nm that is diagnostic for the presence of G4 with antiparallel topology (Fig. 3D). The number of components present in all solutions was further analysed using singular value decomposition (SVD) of the melting profile, described below. The longer diagonal loop (six nucleotides for invTtel-Ttel and telTA-invtelTA) alleviates strain on a parallel G4 topology leading to only parallel G4 for these sequences in K^+^ buffer. Loop length is, therefore, critical to enhance the stability of parallel G4 topology for polarity-inverted sequences over native sequences.

The thermal stability of the resulting G4s was analysed with CD spectroscopy recorded at temperatures in the range of 15–90 °C. Melting temperature (*T*_1/2_) is defined as the temperature at which half of the oligonucleotides are folded into G4s. *T*_1/2_ values (Table 2, Fig. SI-3 and SI-4†) for unmodified sequences in Na^+^ buffer were between 35–40 °C, significantly lower than *T*_1/2_ of letT-Ttel (51 °C), which was expected from the change in molecularity and strand length. Stability of antiparallel G4s with polarity inversions in Na^+^ buffer was even greater than that of letT-Ttel, with *T*_1/2_ in the range 62–76 °C. invTtel-Ttel showed a second melting in Na^+^ buffer at high temperature that was assigned to the parallel G4 species present as the CD peak of positive ellipticity shifted from 255 nm to 265 nm during melting at lower temperatures.

In K^+^ buffer the *T*_1/2_ of all G4s, parallel and antiparallel, was higher than in Na^+^ buffer. Moreover, the *T*_1/2_ values of the polarity-inverted sequences were higher than the *T*_1/2_ of letT-Ttel (58 °C) (Fig. SI-5†), with invtel-tel and invTtel-Ttel having *T*_1/2_ in excess of 83 °C. The sequences telT-invtelT and telTA-invtelTA, which had a lower *T*_1/2_ in Na^+^ buffer than the invtel-tel and invTtel-Ttel, also showed lower *T*_1/2_ in K^+^ buffer. Bimolecular G4s typically have lower thermal stability than unimolecular G4s. This is shown in the increased *T*_1/2_ of letT-Ttel compared to unmodified G4s, but the *T*_1/2_ values of modified G4s are increased further while also maintaining the parallel topology. Both the initial CD results and the *T*_1/2_ measurements show that the presence of inversions encouraged formation of G4s of parallel topology with increased stability. The Ttel and invTtel-Ttel were used to determine if hysteresis occurs for these sequences. In both cases, hysteresis was observed, but the invTtel-Ttel reformed more completely after cooling at the rate of 0.625° min^−1^ (Fig. SI-6†). This fits our expectation of a behaviour for a unimolecular G4 when compared to the bimolecular Ttel sequence.

The number of significant components in the solutions was assessed by applying SVD to the melting data using guidelines proposed by Gray and Chaires.^33^ Briefly,

1 *D* = *USV*^*T*^

*D* is the CD-data matrix where each column represents a CD spectrum at a specific temperature. The column vectors of *U* contain the basis spectra showing the spectral form of each component. These are scaled according to significance according to the singular values in the diagonal matrix *S*. The columns of *V* show the *T*-dependent contribution of each basis spectrum.

The number of components can be assessed based on combined consideration of the: location of an elbow in a plot of the singular values *vs.* component number; relative variance of the singular values; amplitude of the *S*-scaled *U* vectors and decay of the first-order autocorrelation functions of the *U* and *V* matrix columns.^33^ Typical behaviour of systems considered to contain three components and the more prevalent two components are shown in Fig. SI-7 and 8,† respectively. Samples containing two components typically contained the G4 topology indicated in Table 2 and a spectrum corresponding to unstructured DNA. If SVD analysis indicated more than two components were present, the third component was an additional G4 topology with significantly less weight in the overall spectra.

SVD analysis of melting data generally agreed with the above interpretation of CD melting data. The unmodified sequences tel, Ttel and telT were all indicated to contain three components (Fig. SI-7†). The primary component is an antiparallel G4 in Na^+^ buffer and a parallel G4 in K^+^ buffer. The second component was unstructured DNA and the third component in all cases appears to be G4 with the opposite topology, with approximately 10% the weight of the first component. This was not the case for the sequences with inverted polarity and telTA. SVD analysis of these sequences shows only two components, folded and unfolded structures (Fig. SI-8†), with topologies as indicated in Table 2. The primary exception is invTtel-Ttel in Na^+^-containing buffer, which indicates three components and appears to switch from an antiparallel topology to a parallel topology before melting completely. Additionally, telT-invtelT contains a third component, an antiparallel G4, in both Na^+^- and K^+^-containing buffers which is slightly below the 0.8 cutoff value. In general, detailed SVD analysis agrees with the initial analysis of CD spectra indicating that the introduction of polarity inversions stabilises the parallel topology and promotes the shift from three component, mixed G4 topologies to two component, parallel G4 topologies, particularly in K^+^-containing buffers.

### 2.3. A telomeric G4 with a polarity inversion at 5′-end does not form duplexes in the presence of complementary strands

To determine whether inverted polarity prevents duplex formation in the presence of the complementary strand, we focussed on three G4 forming sequences. The first, invtel-tel closely resembles the original bimolecular G4, whereas invTtel-Ttel and telTA-invtelTA have the greatest shift towards the parallel topology according to CD data. The complementary strands were selected to complement both the shorter native strands (12–14 nucleotides) and longer modified strands.

**Fig. 4:**
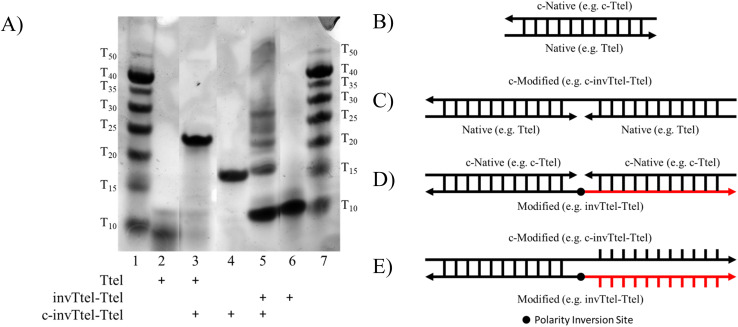
(A) 20% Non-denaturing PAGE showing duplex formation of Ttel and invTtel-Ttel with various complementary sequences (a composite image from a single gel). Lanes 1 and 7: oligothymidylate ladder; lane 2: Ttel; lane 3: Ttel + complementary strand after heating; lane 4: complementary strand c-invTtel-Ttel (control); lanes 5: invTtel-Ttel + complementary strand after heating; lane 6: invTtel-Ttel. See also, Fig. SI-9 in the ESI.† Strand concentration: 100 μM, buffer: 1 × TBE buffer supplemented with 100 mM KCl. Thermodynamic product is formed by mixing a G4 and a complementary sequence and heating the mixture at 90 °C for 5 min, then cooling slowly to 4 °C. (B) Possible products of interaction of native and polarity-inverted DNA sequences with complementary strands: products B and C are canonical duplexes formed by native G-rich DNA (*e.g.* Ttel) and complementary strands (*e.g.* (A) c-Ttel and (B) c-invTtel-Ttel); product D is a possible canonical duplex formed by polarity-inverted G4 (*e.g.* invTtel-Ttel) with two short complementary strands (*e.g.* c-Ttel) arranged in opposite directions; product E shows an expected disruption of a canonical duplex composed of a sequence with inverted polarity (*e.g.* invTtel-Ttel) with a complementary unidirectional strand of the same length (*e.g.* c-invTtel-Ttel).

To evaluate if these G4s resolve to duplex DNA in the presence of their complementary strands (Fig. 4A–E) at room temperature, the G4 was formed then the complementary strand (24–28 nucleotides) added. To elucidate a thermodynamic product, this mixture was heated at 90 °C for 5 min and then slowly cooled down to 4 °C. To evaluate the stability of the G4, these mixtures were then separated by non-denaturing PAGE (Fig. 4A), along with the native and modified G4s and complementary strands as controls.

From Fig. 4A and SI-9† the lanes containing only G-rich sequences (lane 2 for native Ttel and lane 6 for invTtel-Ttel) showed very similar mobilities (corresponding to approx. *T*_10_ marker of the oligonucleotide ladder in lanes 1 and 7) regardless of strand length, which is commonly seen when compact G4s are formed. The results shown in Fig. 4 and SI-9A† suggest that all sequences with inverted polarity could disrupt duplex formation. The unmodified G4 controls, shown in lane 2 on each gel, each had mobility around that of the *T*_10_ marker, with a new band appearing with lower mobility in lane 3 indicating formation of the duplex products (Fig. 4C) in the presence of complementary DNA. These bands are obviously distinct from the native G4 and its complementary strand, indicating formation of a new species. This is most obvious in the Ttel sample with new bands appearing around the *T*_15_ and *T*_25_ markers for short and long complementary sequences, respectively.

In contrast, upon addition of a complementary strand (*e.g.* lane 5 in Fig. 4A), multiple bands, corresponding to the individual components as seen in lanes 4 and 6 of Fig. 4A, were observed, rather than formation of a duplex product such as the one shown in lane 3 of Fig. 4A for the unmodified sequence. Similar gels for telTA-invtelTA and invtel-tel are shown in Fig. SI-9B and C,† respectively. For invTtel-Ttel only faint low retarding bands were observed in the thermodynamic product suggesting that the putative duplexes shown in Fig. 4D and E were not formed. For telTA-invtelTA and invtel-tel (Fig. SI-9B and C,† respectively), formation of duplexes C or D along with individual components were already observed at room temperature (lanes 9 and 11 for telTA-invtelTA and lane 6 for invtel-tel). Duplex products were more dominant after samples were heated and cooled down in the lanes for thermodynamic products. Preliminary CD and non-denaturing PAGE results demonstrate that the use of polarity-inverted nucleotide sequences in G4s inhibited duplex formation and improved G4 stability, particularly for the invTtel-Ttel sequence. However, the bands on native gels could not be quantified to show how effective these modifications were at disrupting duplex formation. Subsequently, ^1^H NMR spectroscopy was used as a method for more accurately ascertaining the secondary structure composition of our DNA mixtures.

For ^1^H NMR analysis we focused on the region from 10.5 to 14.5 ppm, which corresponds to the imino protons of guanosine. The chemical shift of these peaks differs considerably depending on hydrogen-bonding arrangements allowing for secondary structures to be readily distinguished, making this an ideal technique for comparing various secondary structures.^34^ G4s typically have chemical shifts of imino protons around 11–12 ppm, whereas for the canonical Watson-Crick duplex the imino protons appear around 13–14 ppm, and for i-motifs around 15–16 ppm. We also narrowed our initial focus to the invTtel-Ttel sequence which had shown a significant preference for parallel G4 topology, the greatest increase in *T*_1/2_ compared to the unmodified parent sequence, and almost no duplex formation on non-denaturing PAGE in the presence of its complements.

**Fig. 5:**
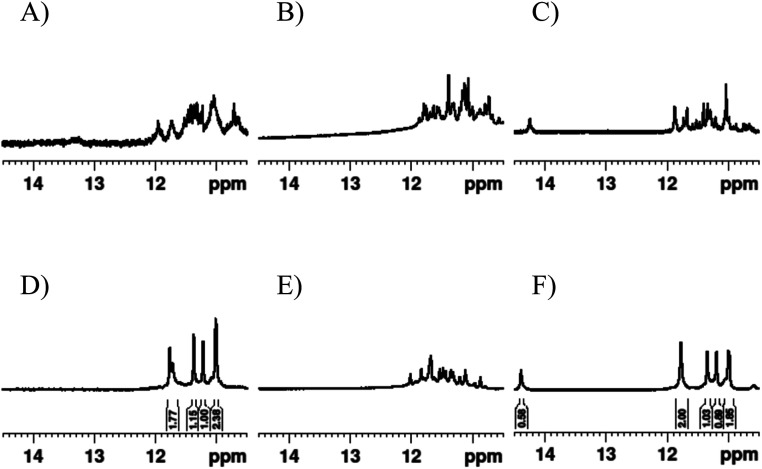
^1^H NMR spectra in the imino proton regions of (A) Ttel, (B) letT-Ttel and (C) invTtel-Ttel in Na^+^-containing buffer; and (D) Ttel, (E) letT-Ttel and (F) invTtel-Ttel in K^+^-containing buffer. Samples contain approx. 200 μM strand concentration, 20 mM sodium phosphate, 10% D_2_O and 1% trimethylsilyl propionate, pH 7.0. K^+^ buffer contains additionally 10 mM KCl.

The ^1^H NMR spectra for Ttel, shown in Fig. 5A and D, was consistent with the expected G4 structure for this sequence. According to the spectra, in Na^+^ buffer a mixture of G4 topologies is present as evident from the appearance of multiple peaks of various intensities at 10.5–12 ppm. Upon addition of K^+^ (as KCl) a topology switch was observed for native Ttel, resulting in the symmetrical G4 reported for bimolecular tel sequences previously.^35^ This is also observed for invTtel-Ttel, suggesting that the polarity inversions did not significantly change the G-tetrad arrangement. On the other hand, the native control letT-Ttel does not exhibit a single conformation, instead forming a mixture of topologies in Na^+^ and K^+^ buffers. Variable temperature experiments were carried out (Fig. SI-10†), which confirm that the G4-containing polarity inversion yields significantly more thermally stable G4 species than the unmodified Ttel G4.

Formation of symmetrical G4s is evident from appearance of distinct imino signals having 2 : 1 : 1 : 2 ratio in integrated signals at 10–12 ppm which is halved for what can be expected for a G4 formed by twelve guanosines. Interestingly, invTtel-Ttel also contains an additional peak at 14.5 ppm, which indicates the presence of Watson–Crick A–T base pairing occurring in the new elongated central loop. This peak is not observed for letT-Ttel, suggesting that this base pairing is only possible with the inversions present. Integration of this signal gives a value of 0.58 which means that there is one A–T base-pair in a G4 with twelve guanines.

**Fig. 6:**
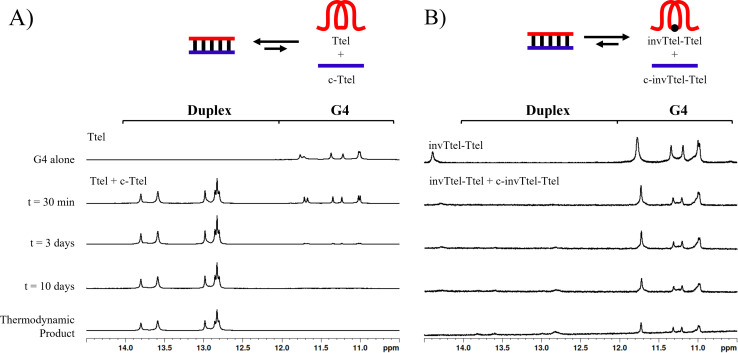
(A) ^1^H NMR spectra in the imino proton region of Ttel mixed with c-Ttel and monitored over time to observe disappearance of a G-quadruplex structure and formation of a canonical duplex. (B) ^1^H NMR spectra in the imino proton region of invTtel-Ttel mixed with c-invTtel-Ttel and monitored over time, observing continued stability of the G4 structure. Strand concentration: 400 μM; buffer: 20 mM sodium phosphate, 10 mM KCl 1% TSP. Peak intensity is consistent relative to internal TSP standard. Thermodynamic product was formed by heating at 90 °C for 5 min, then each sample was cooled slowly to 4 °C.

Next, the duplex-formation experiments analysed by non-denaturing PAGE were replicated using ^1^H NMR spectroscopy. When Ttel and c-Ttel were mixed, as shown in Fig. 6A, peaks began to appear around 13–14 ppm immediately, and after three days almost no peaks were visible in the 11–12 ppm region. Thus, G4 structures began to refold into duplexes almost instantly. Heating of samples followed by slow annealing resulted in a complete switch to the duplex showing that the duplex is both the kinetic and thermodynamic product of mixing these two sequences. However, when invTtel-Ttel and c-invTtel-Ttel were mixed, as shown in Fig. 6B, almost no change was observed, and this was also the case after the mixture was heated and then cooled down. Peaks of low intensity were observable from 13–14 ppm, suggesting that an equilibrium is established, but this equilibrium strongly favours the G4 and not the duplex. One notable shift is the disappearance of the peak at 14.5 ppm, suggesting that the complementary sequence had some interaction with the loop, but doesn't appear to interact with the G4. Overall, these results, on adding (partially) complementary strands establish that the introduction of polarity inversions in G4s significantly diminishes Watson-Crick-duplex formation, thereby increasing the stability of alternative secondary structures such as G4s.

### 2.4. Telomeric G4s containing polarity inversions at 5′-ends remain stable when duplex tails are present

**Fig. 7:**
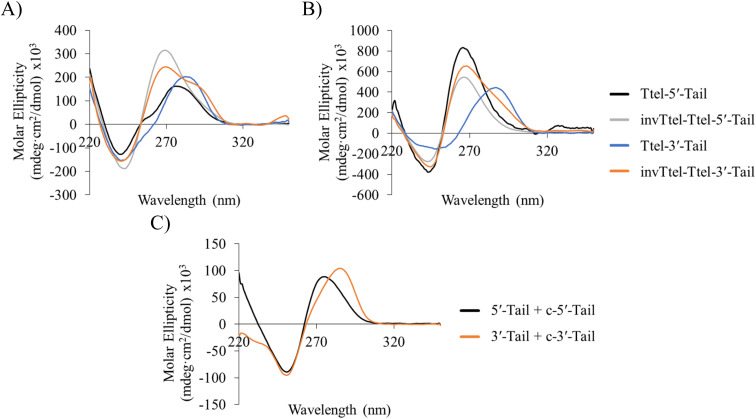
CD spectra of native and modified Ttel sequences with duplex tails based on Widom601 nucleosomal array in (A) Na^+^ buffer and (B) K^+^ buffer at 20 °C. (C) CD spectra of control duplexes composed of the duplex tails. Buffers: Na^+^ buffer consists of 20 mM sodium phosphate, pH 7.0, K^+^ buffer consists of 20 mM sodium phosphate, 10 mM KCl, pH 7.0.

The intended application of these thermodynamically stable G4s required that they are incorporated into longer DNA sequences, as models for G-rich regions in genomic DNA. To accomplish this, we synthesised new sequences containing invTtel-Ttel inserted into the centre of the linker region of the Widom601 nucleosomal array,^36^ incorporating the ends of this region as tails at either end of the invTtel-Ttel sequence (Table 3). Sequence names now include either a suffix of 5′-Tail or 3′-Tail to indicate at which end of the invTtel-Ttel the new duplex forming region is incorporated. Non-complementary *T*_4_ spacers were inserted between the G4 and duplex-forming regions to allow for additional flexibility. CD spectra, shown in Fig. 7A and B, revealed that the native sequences Ttel 5′-Tail and Ttel 3′-Tail correspond to a mixture of structures and topologies resembling those seen for letT-Ttel. In Na^+^ buffer they both most likely adopt antiparallel G4 topologies, but in K^+^ buffer the CD spectra differ. The Ttel 5′-Tail shifts towards a parallel topology, whereas Ttel 3′-Tail remains an antiparallel G4.

**Table 3:** Ttel and invTtel-Ttel sequences with tails for duplex formation

Name	Sequence	Length	G4 topology^a^
Ttel-5′-Tail	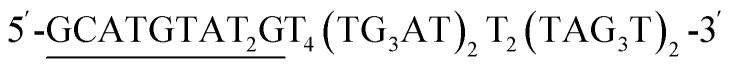	40	Parallel
invTtel-Ttel-5′-Tail	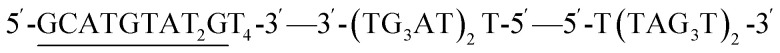	40	Parallel
c-Ttel-5′-Tail	5′-(AC_3_TA)_2_A_2_(ATC_3_A)_2_T_4_CA_2_TACATGC-3′	40	—
Ttel-3′-Tail	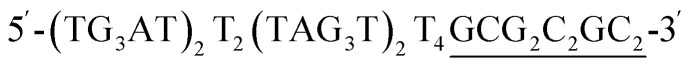	39	Anti-parallel
invTtel-Ttel-3′-Tail	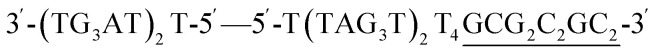	39	Parallel
c-Ttel-3′-Tail	5′-G_2_CG_2_C_2_GCT_4_(AC_3_TA)_2_A_2_(ATC_3_A)_2_-3′	39	—
5′-Tail	5′-GCATGTAT_2_G-3′	10	—
c-5′-Tail	5′-CA_2_TACATGC-3′	10	—
3′-Tail	5′-GCG_2_C_2_GC_2_-3′	9	—
c-3′-Tail	5′-G_2_CG_2_C_2_GC-3′	9	—
Oligo B	5′-TG_3_T_2_G_3_T_2_G_3_T_2_G_3_T_2_G_3_T_2_G_3_T_2_G_3_T_2_G_3_T-3′	40	Parallel
Oligo 2G	5′-TG_3_T_2_AG_3_T_2_AG_3_T_2_AG_3_TG_3_T_2_AG_3_T_2_AG_3_T_2_AG_3_T-3′	45	Antiparallel

a 20 mM sodium phosphate buffer, 10 mM KCl, pH 7.0.

**Fig. 8:**
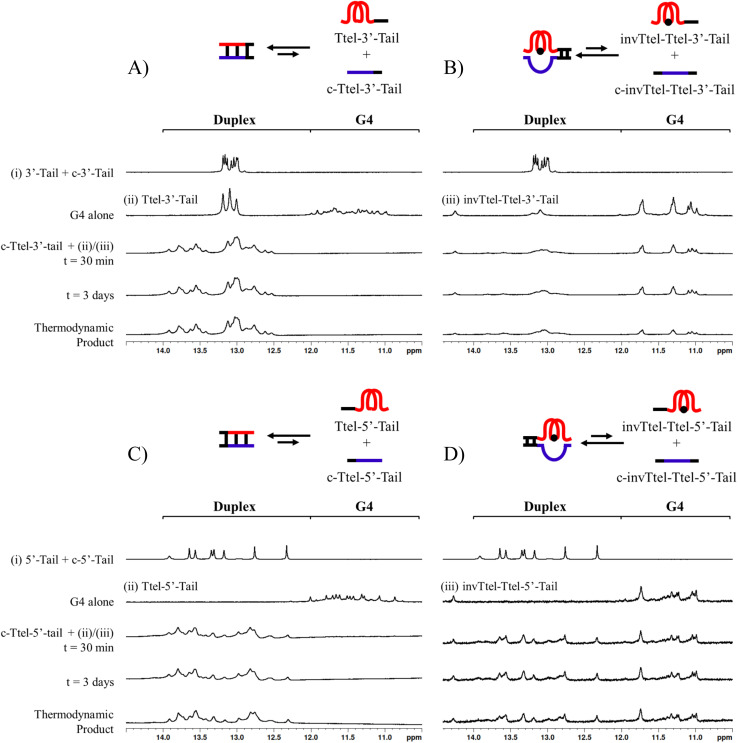
^1^H NMR spectra in the imino region over time with observed disappearance of a G-quadruplex structure and formation of a canonical duplex. (A) Ttel-3′-Tail mixed with c-Ttel-3′-Tail. (B) invTtel-Ttel-3′-Tail mixed with c-invTtel-Ttel-3′-Tail. (C) Ttel-5′-Tail mixed with c-Ttel-5′-Tail. (D) invTtel-Ttel-5′-Tail mixed with c-invTtel-Ttel-5′-Tail. Strand concentrations are 400 μM. Buffer: 20 mM sodium phosphate, 10 mM KCl at pH 7.0. Thermodynamic product was formed by heating the sample at 90 °C for 5 min, then cooling it slowly to 4 °C.

Sequences with inverted polarity favour a parallel G4 topology, even in Na^+^ buffer. In all cases the shoulder is observed at 290 nm, although this is least pronounced for invTtel-Ttel-5′-Tail. This shoulder is possibly caused by the presence of single-stranded DNA, which can give lower intensity CD signals at a range of wavelengths. The sequences added at either end were also tested independently with their complements and were shown to form stable duplexes, as shown by CD spectroscopy in Fig. 7C and ^1^H NMR spectroscopy in Fig. 8.

In ^1^H NMR experiments (Fig. 8, SI-11 and 12†), the unmodified controls do not have the same symmetrical features observed previously for the bimolecular G4s, instead giving spectra more similar to letT-Ttel. The symmetrical structure is still partially preserved in the case of polarity-inverted G4s, although individual peaks in the imino region are not as clearly defined as for the sequences without tails. Overall, this indicates that the original G4 structure was only preserved when inversions were introduced. The 9-mer 3′-Tail has five cytosines which contribute to formation of a self-complementary duplex resulting in distinct peaks at 13–13.5 ppm for G4 alone (Ttel 3′-Tail, Fig. 8A, spectrum ii). This structural feature is suppressed in the polarity-inverted sequence (invTtel-Ttel-3′-Tail, Fig. 8B, spectrum iii). Addition of the complementary strand to native sequences showed similar results to the bimolecular G4s, rapidly unfolding G4 and forming antiparallel duplexes (Fig. 8A and C). In both cases the duplex appears to be both the kinetically and thermodynamically favoured product. Addition of complementary C-rich strands to the polarity-inverted sequences (Fig. 8B and D) showed the appearance of peaks at 12.5–14 ppm, corresponding to duplex formation, but the peaks in the 11–12 ppm range remained mostly unchanged. This indicates that duplexes are formed but comparing these peaks to the tail only controls suggests that duplex formation occurs primarily within the tails. This means that non-G4 regions formed antiparallel duplexes, whereas the polarity-inverted G-rich regions formed stable G4s and duplex formation in this region was disrupted. Heating and slow annealing of these samples also caused very little change in ^1^H NMR spectra, suggesting that the mixed G4/duplex structures are the thermodynamically favoured products.

### 2.5. Hinge region of HP1α can bind telomeric sequences with inverted polarity

**Fig. 9:**
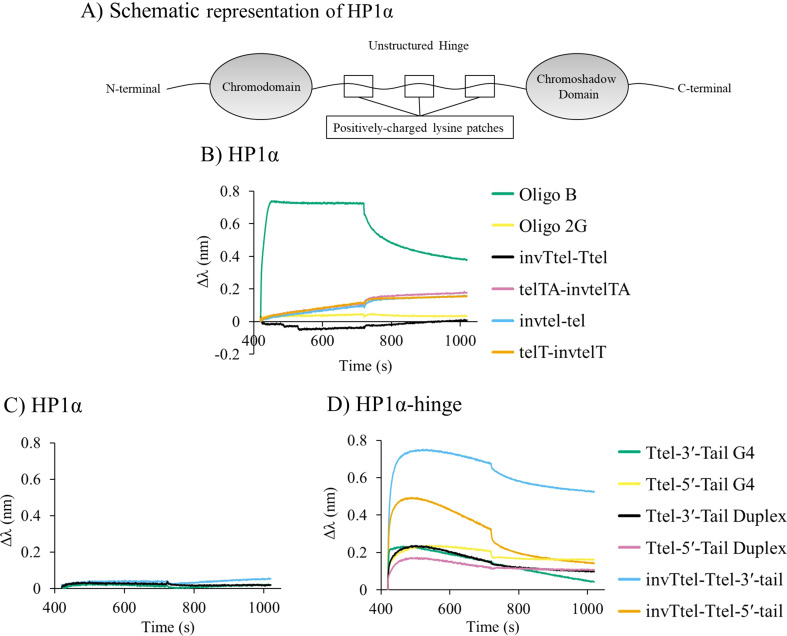
(A) Simplified representation of HP1α structure. Charged patches in unstructured hinge region are proposed to interact with DNA. Biolayer interferometry (BLI) analysis of HP1α binding. (B) Immobilised HP1α to polarity-inverted telomeric G4s compared to parallel and antiparallel G4 controls. (C) Immobilised HP1α to modified Ttel with duplex-forming tail at the 3′-end compared to unmodified Ttel G4s and duplexes. (D) Immobilised HP1α hinge domain to modified Ttel with tails compared to unmodified Ttel G4s and duplexes. Conditions: 2 μM strand concentration, protein concentrations are 100 μg mL^−1^ HP1α and 33 μg mL^−1^ HP1α hinge ; buffer: 20 mM sodium phosphate, 10 mM KCl, pH 7.0.

Chromatin remodelling protein HP1α (Fig. 9A) is known to bind to only to (quasi-)native G4s of parallel topology.^3^ Therefore, HP1α was chosen to investigate whether, or not, this highly selective G4 binder would bind to parallel G4s with inverted topology. Biolayer interferometry (BLI) was performed where his_6_-tagged HP1α was immobilised on nickel sensor tips and immersed in solutions containing oligonucleotides with polarity inversion. We investigated the HP1α-G4 complex formation with G4s having duplex tails, as well as the Oligo B (parallel G4) and Oligo 2G (antiparallel G4) controls shown in Table 3.

As previously shown,^3^ HP1α binds to the parallel G4 formed by Oligo B but not to the antiparallel G4 (Oligo 2G), as shown in Fig. 9B. To our surprise, wild-type HP1α also showed little to no affinity for modified (polarity-inverted) G4s of parallel topology either before (Fig. 9B) or after (Fig. 9C) inclusion of a duplex forming tails.

Since HP1α′s charged lysine patches at residues 89–91 and 104–106 located in the unstructured hinge region (Fig. 9A) have been implicated in G4 binding we decided to test if the HP1α hinge binds to our synthetic constructs. A his_6_-tagged hinge of HP1α (Fig. SI-13†) was recombinantly expressed in *E. coli* and purified. The hinge was also immobilised on nickel sensor tips, and the tips then immersed in a K^+^ solution containing a G4 with a polarity-inverted sequence or a control native G4 or a duplex. In this case, we observed strong binding of the HP1α hinge domain for G4s with a polarity-inverted sequence (Fig. 9D). Table 4 shows the dissociation constants, *K*_D_, which were highest for duplexes (*i.e.* very weak binding), followed by the native G4s, but, surprisingly, polarity-inverted G4s showed almost a five-fold increase in binding affinity over unmodified G4s (smallest *K*_D_). This suggests that lysine patches in the unstructured hinge region of HP1α facilitate binding to unmodified and polarity-inverted G4s whereas structured chromo- and chromoshadow domains are responsible for the selectivity of wild-type HP1α binding to G4s.

**Table 4:** Biolayer interferometry data for binding of the hinge of HP1α to polarity-inverted G4s

Name	Sequences	*k*_on_/(1000 M^−1^ s^−1^)	*k*_off_/(10^−3^ s^−1^)	*K*_D_ (nM)
3′-Tail G4 control	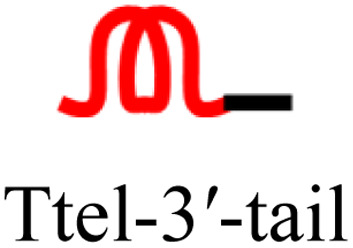	85.6 (19)^a^	0.92 (11)	10.8 (13)
5′-Tail G4 control	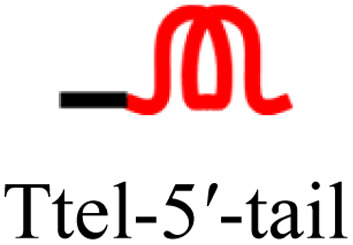	39 (3)	15.4 (7)	390 (3)
3′-Tail duplex control	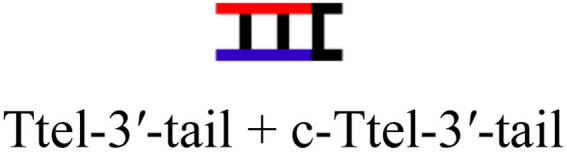	64 (8)	53 (4)	840 (13)
5′-Tail duplex control	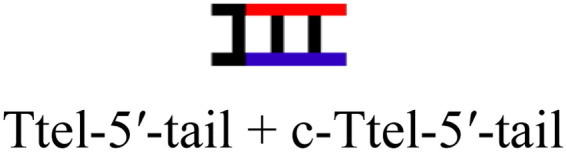	32 (11)	28 (3)	900 (3)
3′-Tail Inv-G4	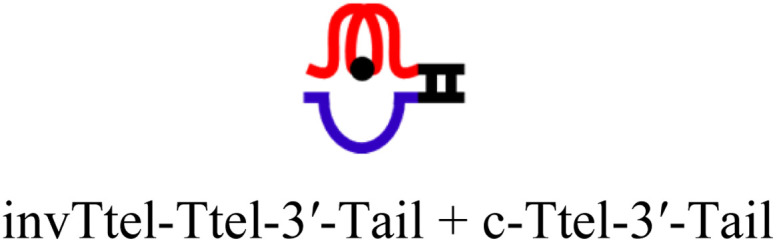	85.2 (9)	4.85 (9)	56.9 (12)
5′-Tail Inv-G4	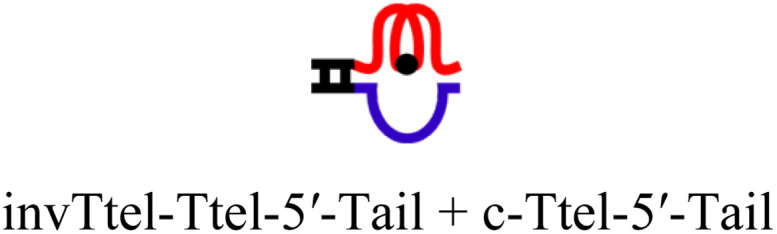	149 (9)	39.1 (15)	263 (19)

a The error (estimated standard deviation) in the least significant digit(s) is provided in parentheses.

We proposed that several antiparallel G4s did not bind to HP1α due to the presence of lateral or diagonal loops blocking binding sites.^3^ We tentatively conclude that the location of the introduced polarity inversions creates an additional diagonal loop, causing the modified sequences to behave similarly to antiparallel G4s with respect to HP1α binding. Our telomeric G4s based on polarity-inverted sequences form a symmetrical G4 of parallel topology but all of them have a diagonal or lateral loop that shields one of the external G-tetrads (seen in Fig. 2D and E). This feature, which is typical for antiparallel G4s, might prevent our polarity-inverted G4 constructs from interacting with HP1α. We also infer that HP1α selectivity may be determined not just by G4 topology, but also by the specific loop arrangements seen primarily in antiparallel G4s.

## 3. Conclusions and perspectives

Internal polarity inversions introduce several unique properties to oligonucleotides. Several DNA sequences containing inversions favoured parallel over antiparallel topologies and had higher thermal stability than native oligonucleotides. The sequences with inverted polarity did not form duplexes in the presence of the complementary C-rich strands, instead remaining as thermodynamically stable G4s. When incorporated into longer DNA sequences the G4s remained thermodynamically stable even when the remainder of the sequence formed a duplex. This suggests that polarity-inverted nucleotides could be used to incorporate thermodynamically stable G4s into larger duplex DNA complexes.

We also explored the binding of these modified DNA strands to a known G4-binding protein, HP1α, an essential protein for chromatin maintenance. The polarity inverted DNA molecules did not bind to wild-type HP1α, but formed complexes with the isolated hinge region that connects the two structured domains of HP1α. This hinge region lacks some of the specificity of the wild-type protein, suggesting that the introduction of an additional loop, characteristic of antiparallel G4s, may explain the lack of binding of the wild-type HP1α to the modified species. However, the affinity of the hinge region suggests that parallel G4-forming sequences with polarity inversion do not inherently disrupt protein interactions. Some proteins have been shown to bind to antiparallel G4s, such as POT1, which showed specificity for antiparallel structures, or SP1, which bound both parallel and antiparallel G4s.^12^ The interactions of G4-binding proteins such as these may not be disrupted by the introduction of lateral or diagonal loops in the same way as HP1α, and their interactions with sequences containing inversions are worth investigating. Furthermore, these experiments demonstrate the ability of polarity-inverted nucleotides to stabilise G4s and disrupt duplex formation. These results could be applied to other known G4-forming sequences, obtaining G4s with similar properties without introducing undesirable structural elements.

The potential uses for this type of modified sequence are extensive. The initial intended application is to construct large DNA structures containing thermodynamically stable G4s. This would allow for better modelling of DNA as it exists in cells while ensuring the presence of thermodynamically unfavourable secondary structures (such as G4s) during longer duration experiments, such as NMR or small-angle X-ray scattering (SAXS). Other applications include those explored previously, both for polarity-inverted nucleotides and modified DNA in general. Increased stability of G4s could be used to inhibit the activity of proteins whose function is dependent on duplex DNA, a possibility that has already been explored in the context of G4-binding ligands.^37,38^ Development of better DNA-based inhibitors could be improved by using polarity-inverted nucleotide sequences. Similarly, the affinity of DNA aptamers could be improved through the use of broader nucleotide libraries, potentially including polarity-inverted nucleotides. The data presented here serve to demonstrate the effectiveness of these nucleotides for stabilising non-canonical, thermodynamically unfavourable DNA secondary structures in short sequence while in the presence of complementary DNA. Further experimentation is necessary to determine the implications of these results on the development of new DNA technologies.

## 4. Experimental section

The oligonucleotide controls used in this study were obtained from Integrated DNA Technologies. These sequences were dissolved in Milli-Q water and G4 structures were formed by diluting the stock solutions in 20 mM NaH_2_PO_4_/Na_2_HPO_4_ buffer. Potassium chloride (10 mM) was added when required. Samples were annealed by heating at 90 °C for 5 min, cooling slowly to 4 °C and storing for 24 hours at 4 °C.

DNA synthesis was carried out using a Mermade 4 DNA/RNA automated synthesiser. Controlled pore glass supports carrying the first nucleoside were obtained from Dnature Diagnostics and Research Ltd (New Zealand). Standard 5′-O-DMT-3′-O-phosphoramidites of nucleosides were obtained from Innovassynth Technologies (I) Ltd (India) and inverted 3′-*O*-DMT-5′-*O*-phosphoramidites were obtained from Chemgenes Corporation (USA). The coupling time of inverted phosphoramidites was increased to 10 min. After the synthesis, oligodeoxynucleotides were cleaved from the solid support with 28% aq. ammonia at 55 °C for 12 hours. Ammonia was evaporated using a speed-vac Eppendorf Concentrator Plus and oligodeoxynucleotides were purified using Thermo Scientific UltiMate 3000 UHPLC with an Alltech 250 mm × 4.6 mm, 10 μm Hypersil Gold column (Reverse-Phase) or a TSKgel SuperQ-5PV 7.5 mm I.D. × 7.5 cm, 10 μm column (Ion-Exchange). Purified oligodeoxynucleotides were desalted using NAP-5 size exclusion columns. Sequences were verified using mass spectrometry recorded in 15% methanol/H_2_O using electrospray ionisation MS with a Thermo Scientific Q Exactive Focus Hybrid Quadrupole-Orbitrap Mass Spectrometer. Masses are reported in atomic mass units (a.m.u.).

Folded oligonucleotides (100 μM) were separated on a 20% polyacrylamide gel (PAGE) in 1× TBE buffer (90 mM Tris-Borate and 2 mM Na_2_H_2_EDTA, pH 8.3), with either 100 mM KCl or NaCl added, at 4 °C. A 20% aq. glycerol solution was added to increase sample density for loading. After electrophoresis (5–10 W), the gel was rinsed with H_2_O, stained with 0.35% Stains-All (Merck) in 50% formamide : H_2_O for 15 min, destained in H_2_O, and then imaged. 20% denaturing PAGE was prepared in 1 × TBE Buffer (pH 8.3) with 7 M urea. For denaturing PAGE, oligodeoxynucleotide samples were heated at 90 °C for 5 min and then cooled to room temperature prior to loading on the gel.

Circular dichroism (CD) spectra were recorded using a Chirascan CD spectrophotometer (150 W Xe arc) from Applied Photophysics with a Quantum Northwest TC125 temperature controller. Oligodeoxynucleotides were diluted to 10 μM in the reported buffers. Scans were taken over a range of 220–350 nm with a bandwidth of 1 nm and response of 0.25 s. A buffer-only baseline was subtracted from each CD spectrum before they were smoothed by averaging 10 neighbour points using software provided by Applied Photophysics Ltd. Melting experiments were performed by recording CD spectra every 2.5 °C with equilibration for 2.5 min at each temperature from 15 to 90 °C. The signal at maxima and minima was assessed and values were converted to fraction folded (*θ*_T_) using the formula:*θ*_T_ = (*θ* − *θ*_min_)/(*θ*_max_ − *θ*_min_)

*θ* is the CD signal at that temperature while *θ*_max_ and *θ*_min_ are the signal when completely folded and completely unfolded, respectively. *T*_1/2_ is the temperature at which half of the structure is unfolded (*θ* = 0.5). *T*_1/2_ is not reported for sequences which did not completely unfold within the specified temperature range. For SVD analysis, raw data were truncated outside the range 225 to 340 nm to remove artifacts. Baseline correction was performed by subtracting the average intensity in the range 320–340 nm from each spectrum. Data were smoothed using the algorithm using a Savitzky–Golay filter with a 9-point window and polynomial order of 2. The script written in Python is provided in ESI† along with instructions for using it on https://www.Jupyter.org.

^1^H NMR spectra were recorded using a Bruker 700 MHz spectrometer using trimethylsilylpropanoic acid (TSP) as an internal standard. Chemical shifts are reported in parts per million (ppm) relative to the TSP methyl signal at 0.00 ppm. Spin multiplicities are described as: s (singlet), br.s. (broad singlet), d (doublet), dd (doublet of doublets), t (triplet), q (quartet), m (multiplet). Coupling constants are reported in Hertz (Hz).

Biolayer interferometry (BLI) using a BLItz system (ForteBio, USA) was used to examine the binding of HP1α to a range of oligodeoxynucleotides as indicated at room temperature. Ni-NTA biosensors (ForteBio) were hydrated with 1 × interaction buffer (IB) containing 100 mM KCl, 50 mM NaCl, 20 mM NaH_2_PO_4_/Na_2_HPO_4_, pH 8.0. G4s were folded in the same buffer using the method described above. 4 μL of 100 μg mL^−1^ his-tagged HP1α was used to load the Ni-NTA biosensor for 5 min to reach ∼4 nm of signal. The biosensor was then washed with 1 × IB. The association step was performed using 2 μM solutions of oligodeoxynucleotides prepared in 1 × IB or just 1 × IB (reference) for 5 min, then the dissociation step was performed using 1 × IB for 5 min. Reference runs were subtracted from test runs to account for dissociation of the protein. Oligodeoxynucleotides were tested for interaction with Ni-NTA biosensor tips prior to experiments. BLItz Pro 1.2 software was used for curve fitting and *K*_D_ calculations.

## Data availability

Original data are available upon reasonable request from the authors.

## Author contributions

B. C., G. B. J., T. K. H. and V. V. F. designed the research, B. C. performed the synthesis and purification of oligodeoxynucleotides, CD and PAGE experiments; R. J. R. expressed and purified proteins and performed BLItz experiments. P. J. B. E. and B. C. performed NMR experiments. P. J. B. E. wrote the SVD Python script and B. C. performed the analysis of melting curves. All authors analysed the data; B. C. wrote the first draft of the article, all authors have read and agreed to the published version of the manuscript.

## Conflicts of interest

Authors declared no conflict of interests.

## Supplementary Material

SC-OLF-D3SC05432B-s001

SC-OLF-D3SC05432B-s002
