# Supplementary material for: Inverted strand polarity yields thermodynamically stable G-quadruplexes and prevents duplex formation within extended DNA
Source: Chem Sci. 2024 Aug 27;15(37):15417–31. doi: 10.1039/d3sc05432b (PMC11376080; doi:10.1039/d3sc05432b)
Supplement: SC-015-D3SC05432B-s002 [file SC-015-D3SC05432B-s002.pdf]

Supplementary Information (SI) for Chemical Science.
This journal is © The Royal Society of Chemistry 2024

Supporting Information for

Inverted Strand Polarity Yields Thermodynamically Stable G-quadruplexes and Prevents Duplex Formation within Extended DNA

Bruce Chilton, Ruby J. Roach, Patrick J. B. Edwards, Geoffrey B. Jameson, Tracy K. Hale and Vyacheslav V. Filichev*

School of Food Technology and Natural Sciences, Massey University, Private Bag 11–222, Palmerston North 4442, New Zealand

A) invtel-tel

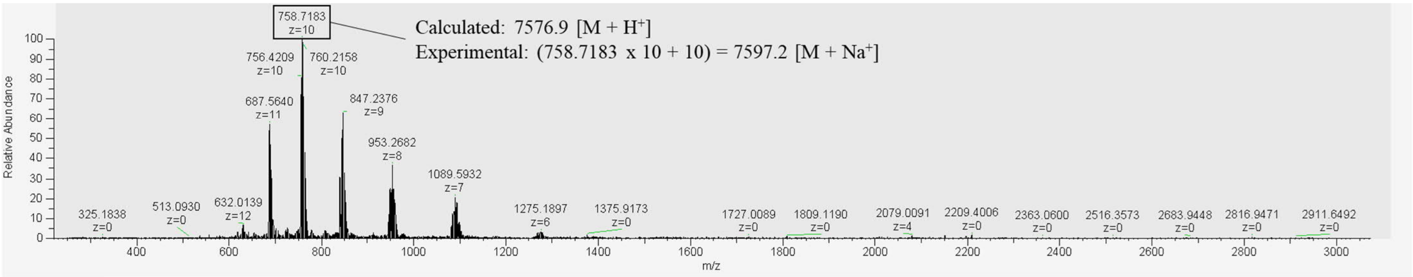

B) invTtel-Ttel

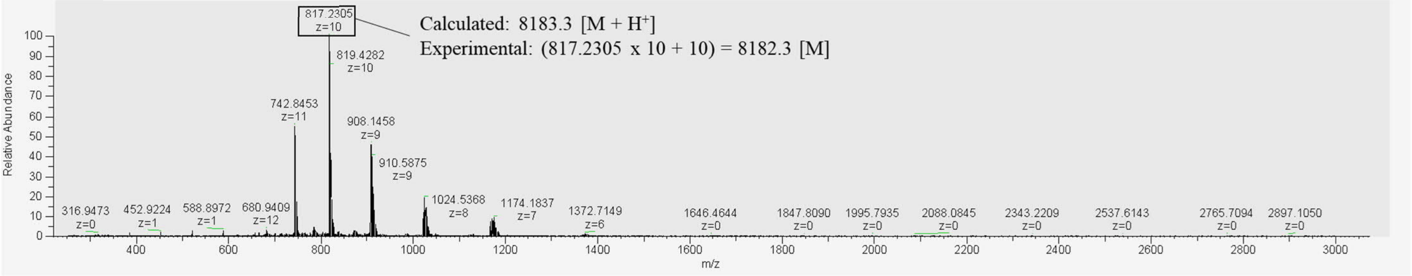

C) telT-invtelT

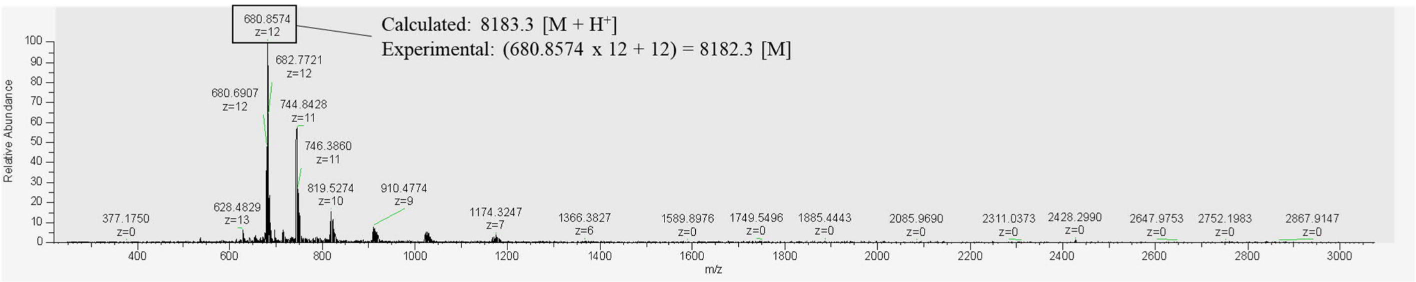

D) telTA-invtelTA

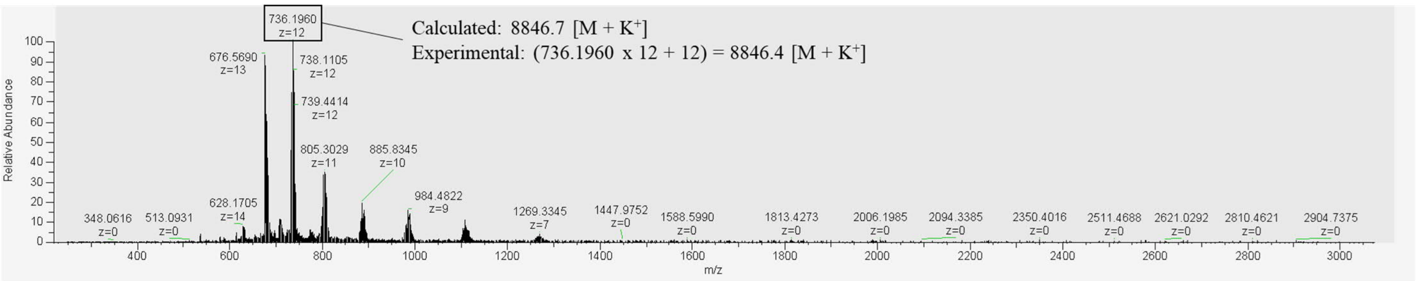

E) invTtel-Ttel-3′-tail

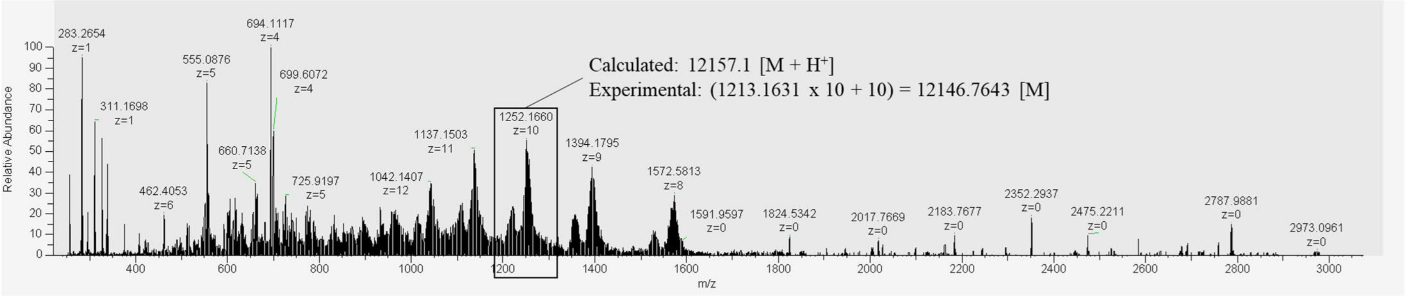

F) invTtel-Ttel-5′-tail

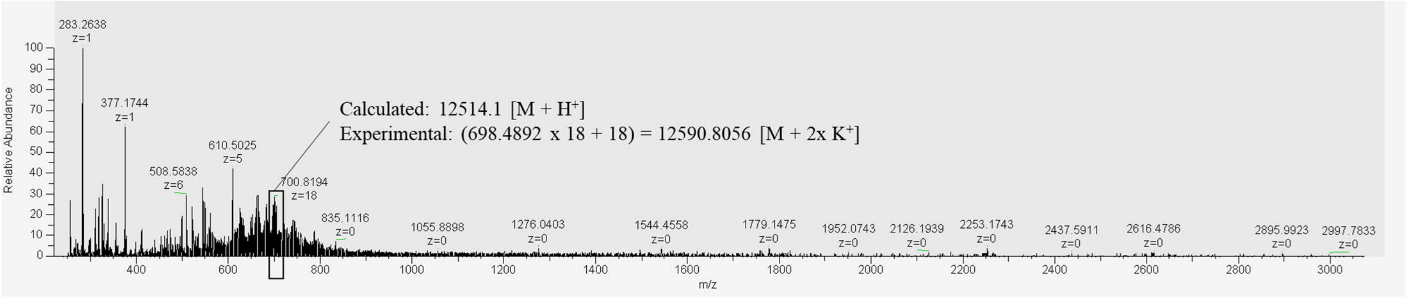

Figure SI-1. Mass spectrometry of synthesised DNA sequences with example calculated masses. (A – D) Polarity-inverted sequences provided in Table 1. (E – F) Polarity-inverted sequences with tails provided in Table 3. The larger size of these sequences results in a greater number of adducts appearing in the spectrum. Conditions: 20 µM strand concentration, 15% methanol in milli-Q water.

Table SI-1: Example of mass calculations for invtel-tell:

Peak	Charge (q)	Experimental Mass (Peak x q + q)
1089.5932	+7	7634.1524 [M + Na^+ + K^+]
953.2682	+8	7634.1456 [M + Na^+ + K^+]
847.2376	+9	7634.1384 [M + Na^+ + K^+]
756.4209	+10	7574.206 [M]
758.7183	+10	7597.183 [M + Na^+]
760.2158	+10	7612.158 [M + K^+]
687.5640	+11	7574.204 [M]

The peaks highlighted are the ions that are detected most frequently. Peaks with identical charges provide masses corresponding to the calculated mass with different ratios of Na^+ and K^+. Peaks with different charges provide the same mass (the calculated mass is synthesis was successful. For accurate calculations, multiple peaks with each charge are used to determine that the correct experimental mass and its corresponding salts are present.

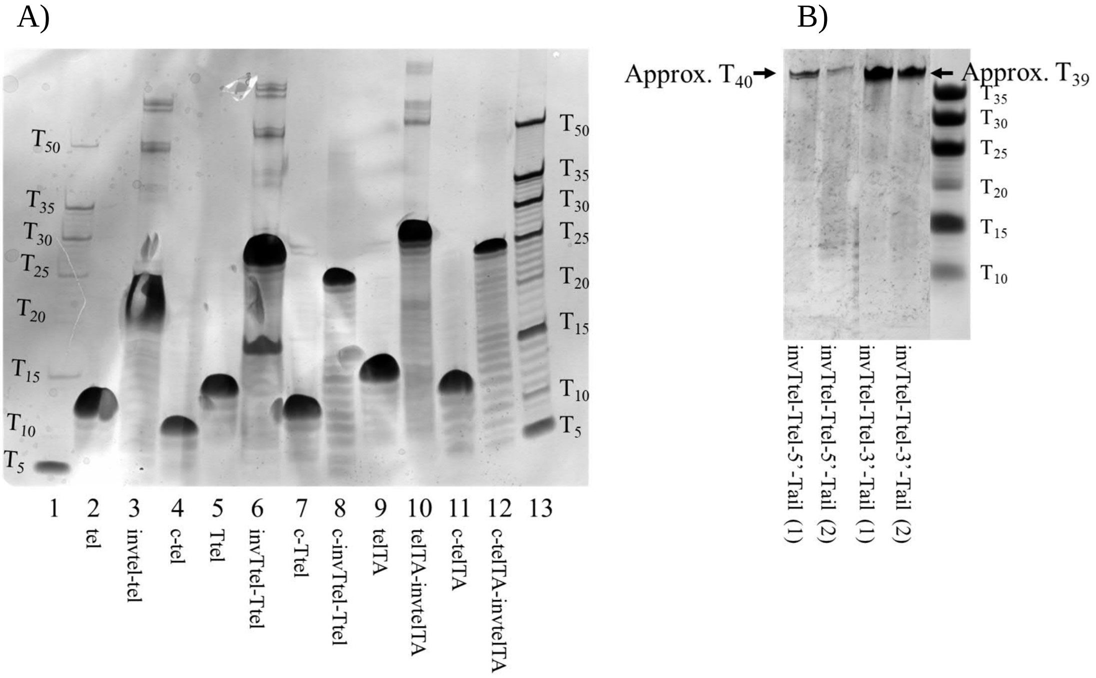

Figure SI-2. 20% denaturing PAGE used to confirm length of both modified and native G4-forming sequences. (A) Unmodified and polarity-inverted telomeric sequences (Table 1). (B) Synthesised polarity-inverted sequences with duplex forming tails (Table 2). Conditions: TBE Buffer with 7 M urea added, samples heated at 90 °C prior to loading on gel.

A) tel

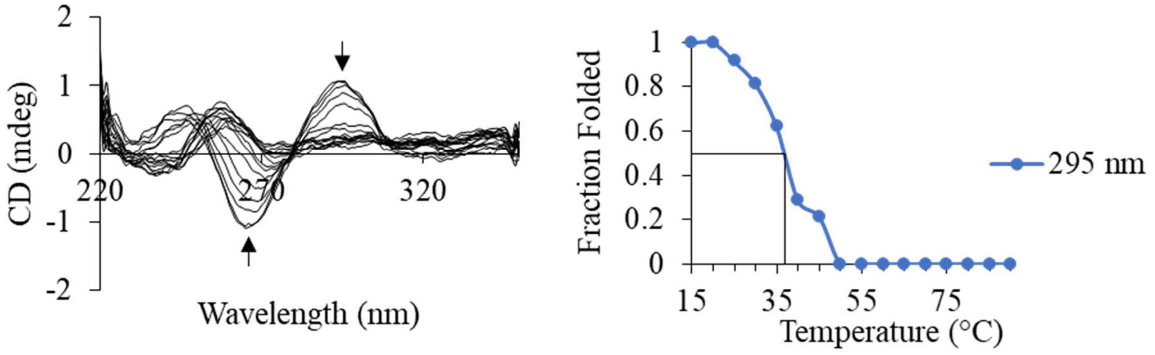

B) Ttel

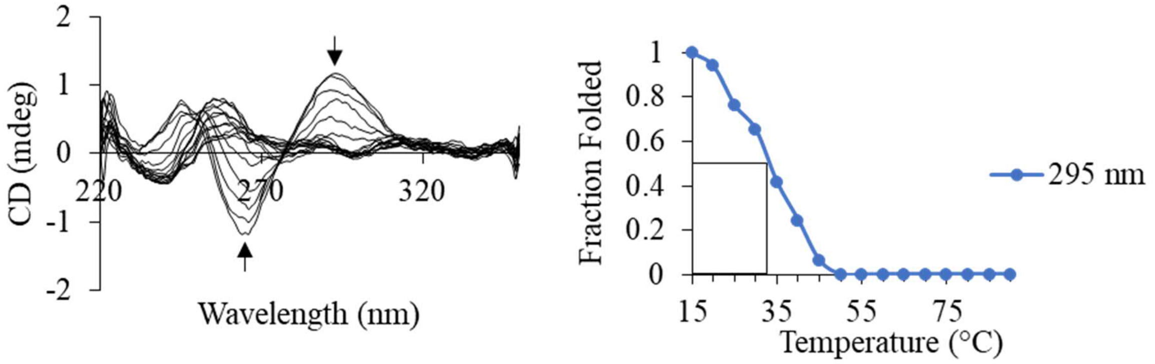

C) telT

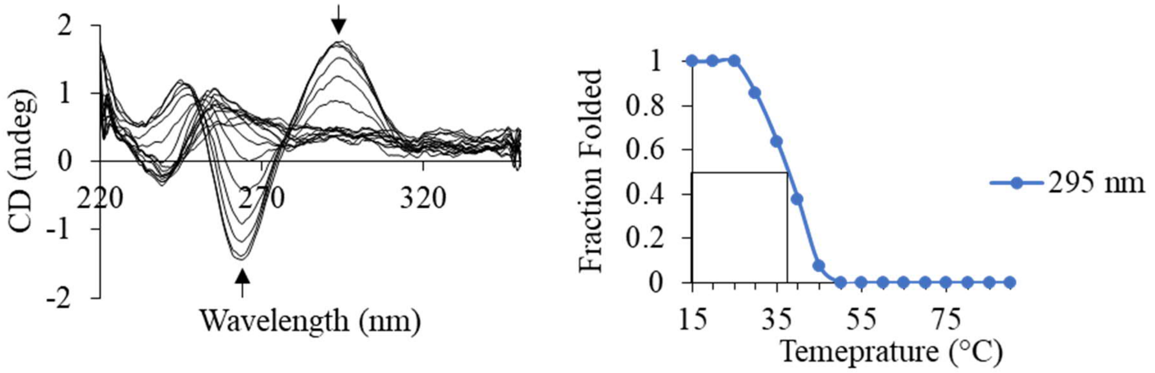

D) telTA

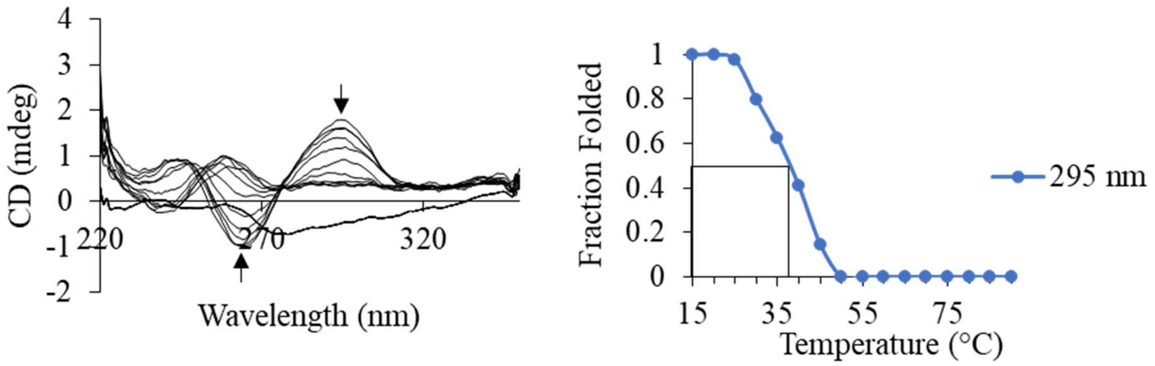

E) invtel-tel

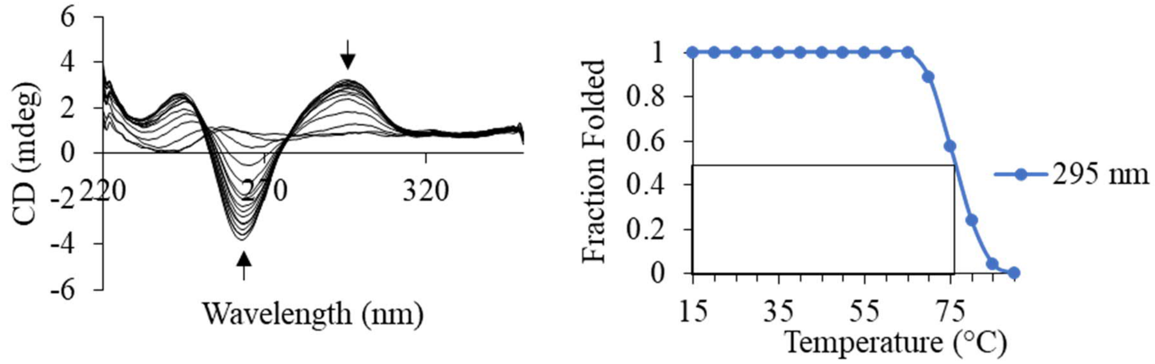

F) invTtel-Ttel

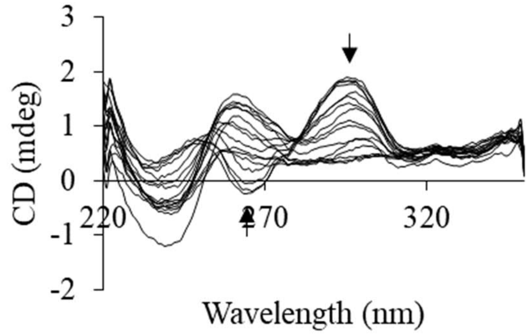

No clear melting profile

G) telT-invtelT

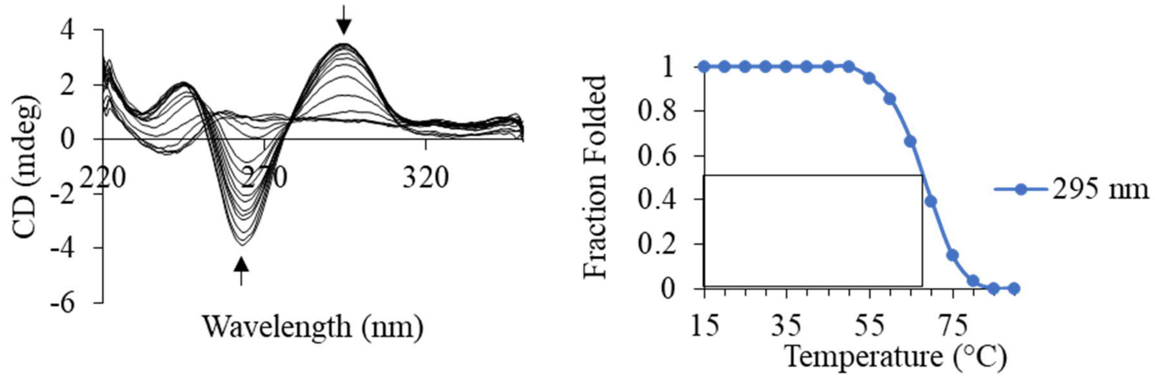

H) telTA-invtelTA

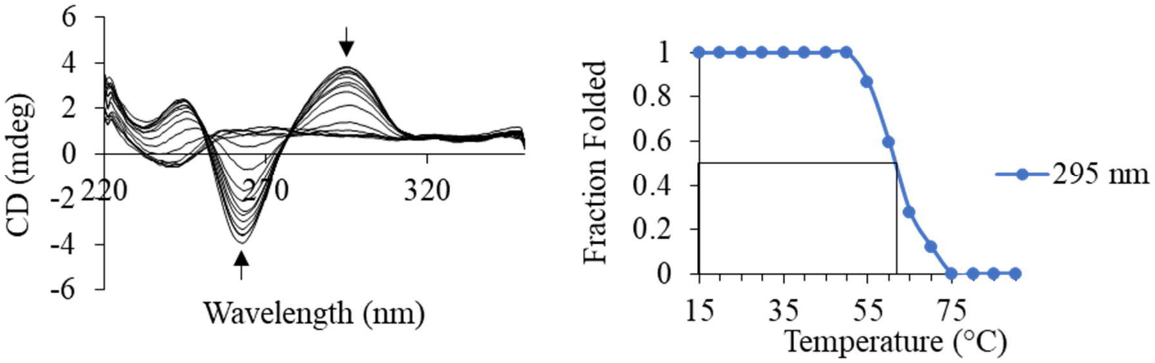

Figure SI-3. CD melting spectra of telomeric sequences in Na^+ containing buffer. (A – D) Unmodified, bimolecular G4 structures. (E – H) Polarity-inverted, unimolecular G4 structures. Conditions: 10 mM Lithium cacodylate, 100 mM NaCl, pH 7.2, heated to 90 °C at 0.75 °C/min.

A) tel

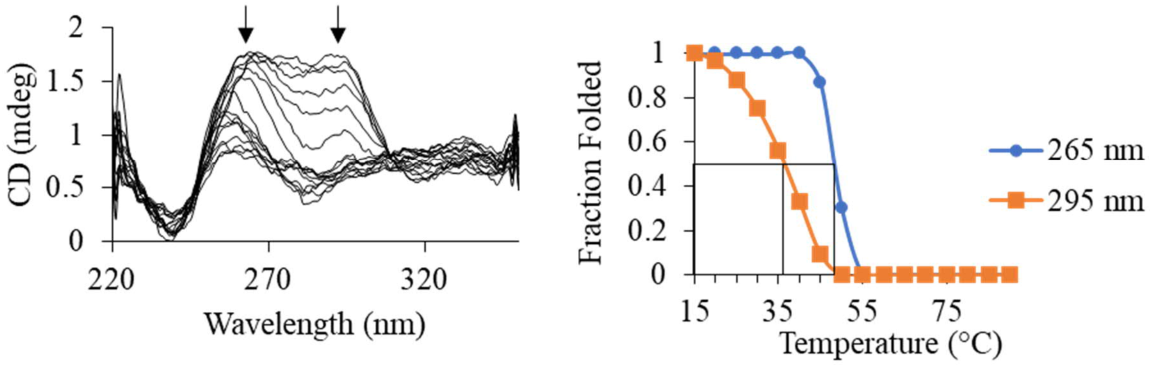

B) Ttel

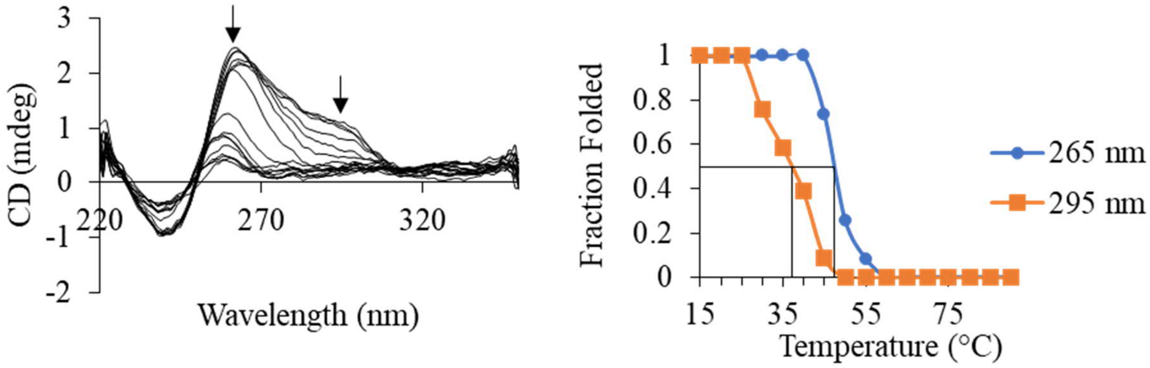

C) telT

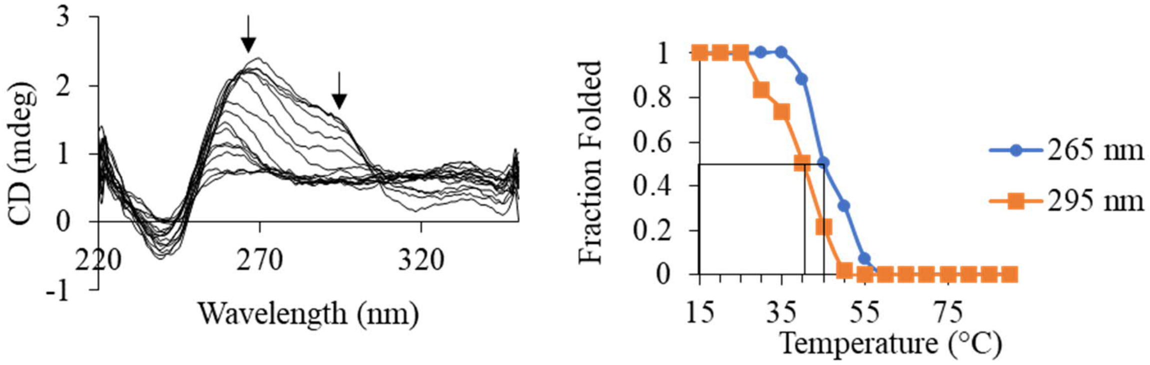

D) telTA

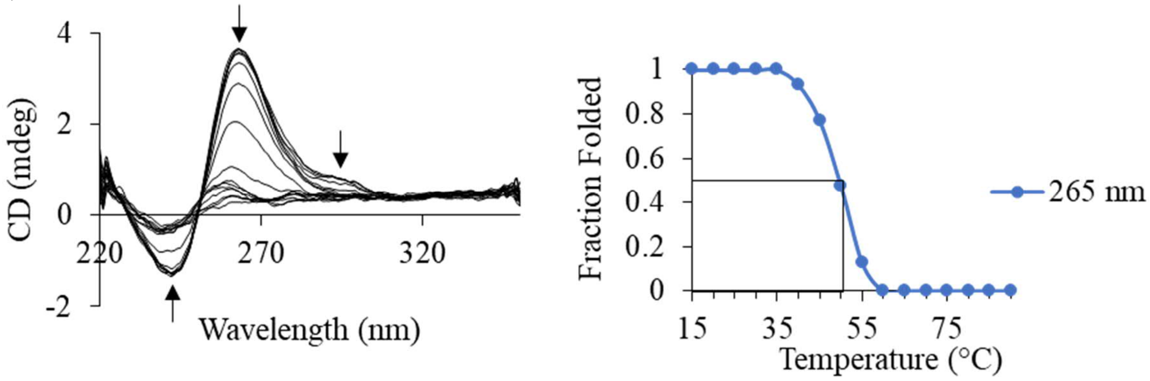

E) invtel-tel

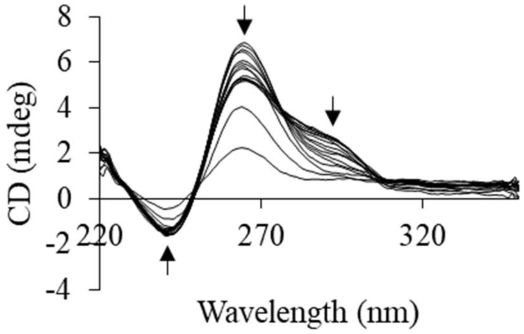

Not fully melted at 90 °C

F) invTtel-Ttel

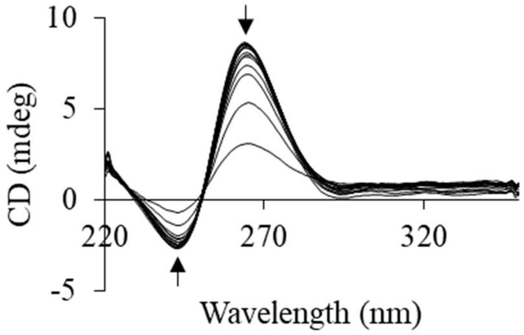

Not fully melted at 90 °C

G) telT-invtelT

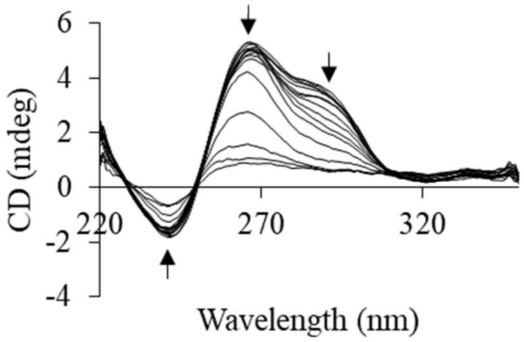

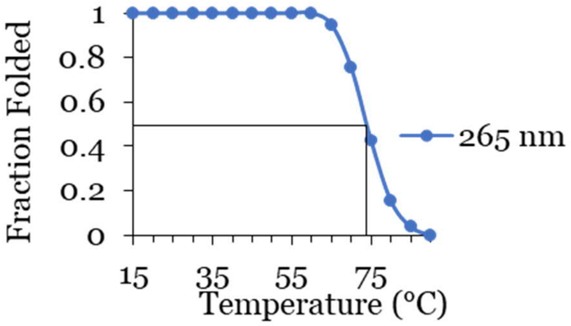

H) telTA-invtelTA

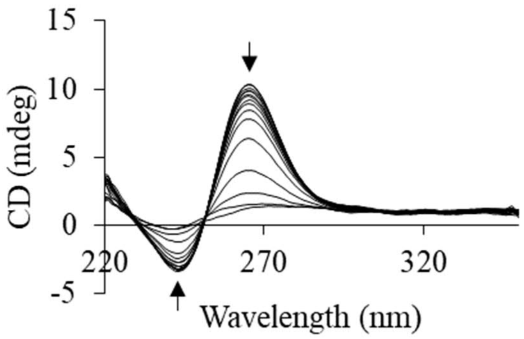

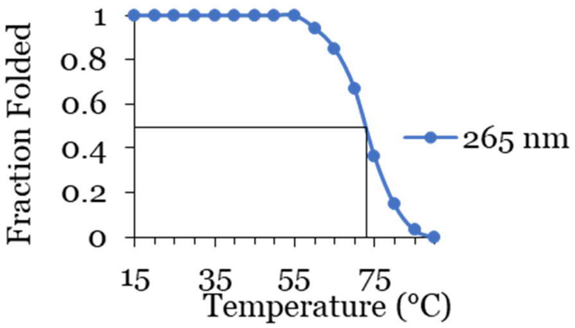

Figure SI-4. CD melting spectra of telomeric sequences in K^+ containing buffer. (A – D) Unmodified, bimolecular G4 structures; (E – H) Polarity-inverted, unimolecular G4 structures. Conditions: 10 mM lithium cacodylate, 100 mM KCl, pH 7.2, heated to 90 °C at 0.75 °C/min.

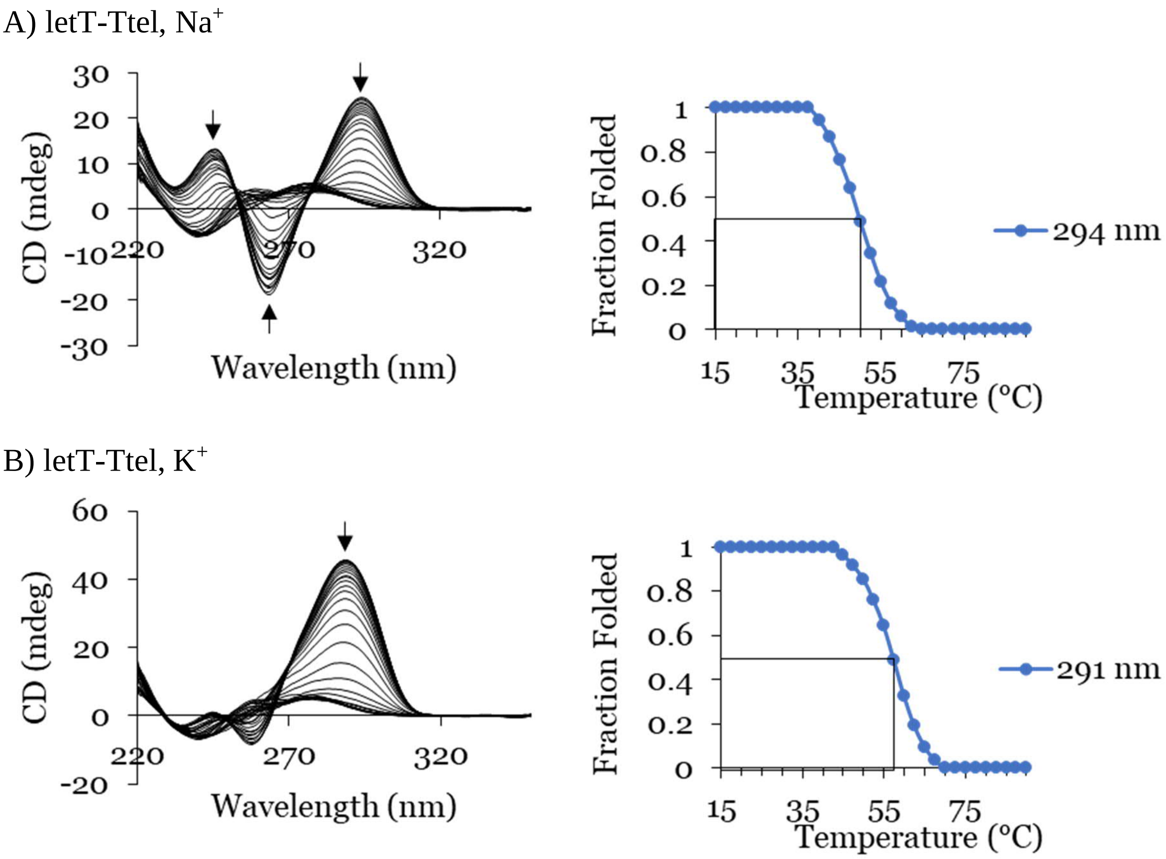

Figure SI-5.CD melting spectra of Ttel-2rev control in Na^+ and K^+ containing buffers. (A) 10 mM lithium cacodylate, 100 mM NaCl, pH 7.2, heated to 90 °C at 1 °C/min. (B) 10 mM lithium cacodylate, 100 mM KCl, pH 7.2, heated to 90 °C at 0.625 °C/min.

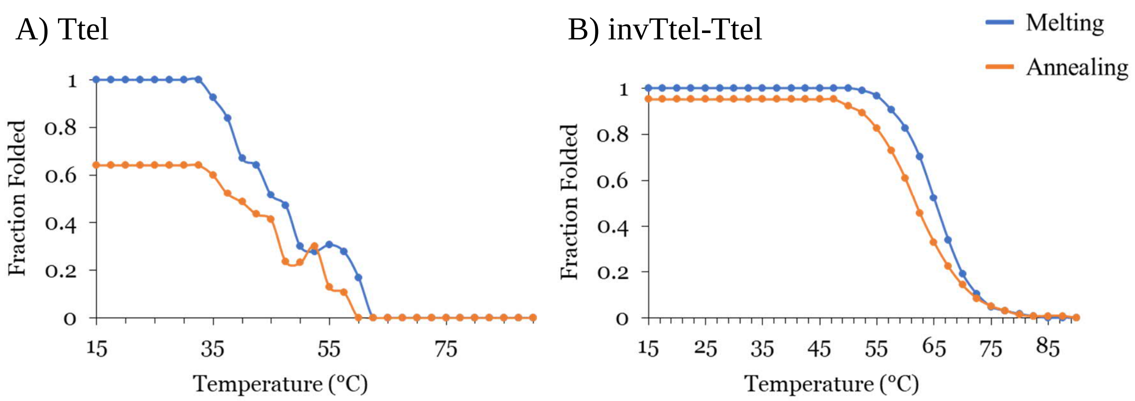

Figure SI-6. CD melting spectra of A) Ttel (260 nm) and B) invTtel-Ttel (264 nm), including annealing. Conditions: 10 µM sequence concentration, 10 mM lithium cacodylate, 100 mM KCl, pH 7.2, heating/cooling rate is 0.625 °/min.

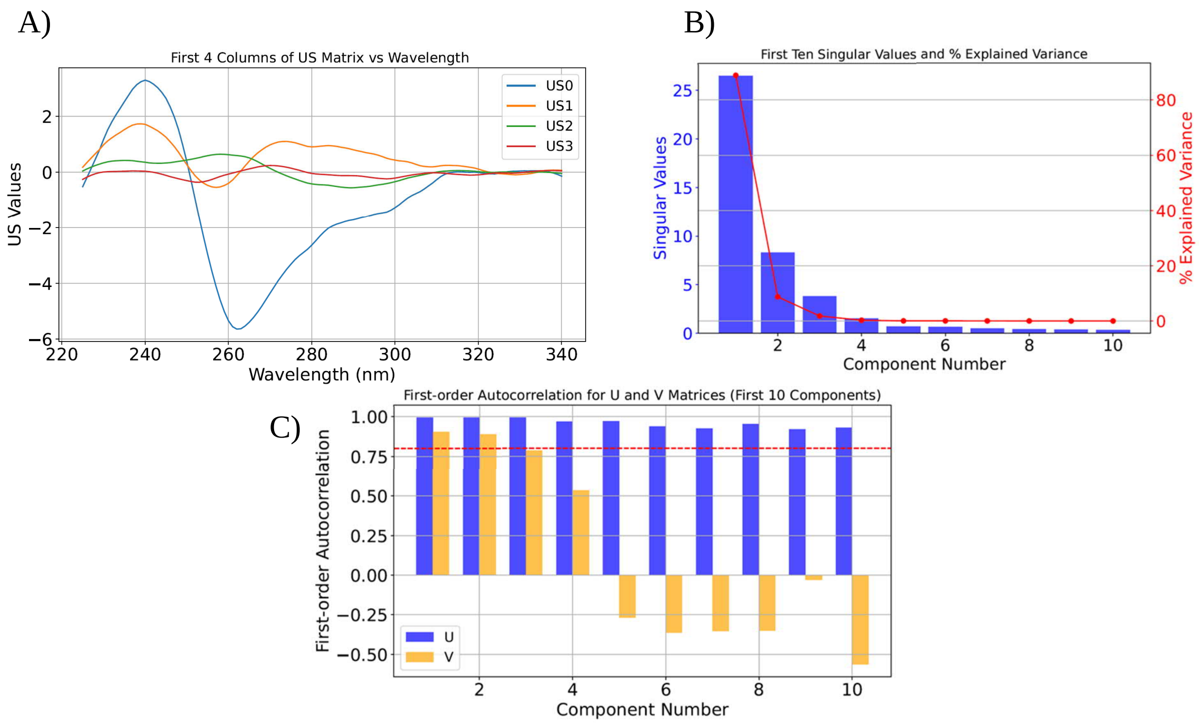

Figure SI-7. Example of singular value decomposition analysis for Ttel in K^+ containing buffer containing three components assessed on the criteria of Gray and Chaires. A) Basis spectra of first four components weighted by their singular values. B) First 10 singular values (left-hand axis) and their contribution to the relative variance (right-hand axis). C) First-order autocorrelation of U and V columns using the suggested cutoff of ~0.8.

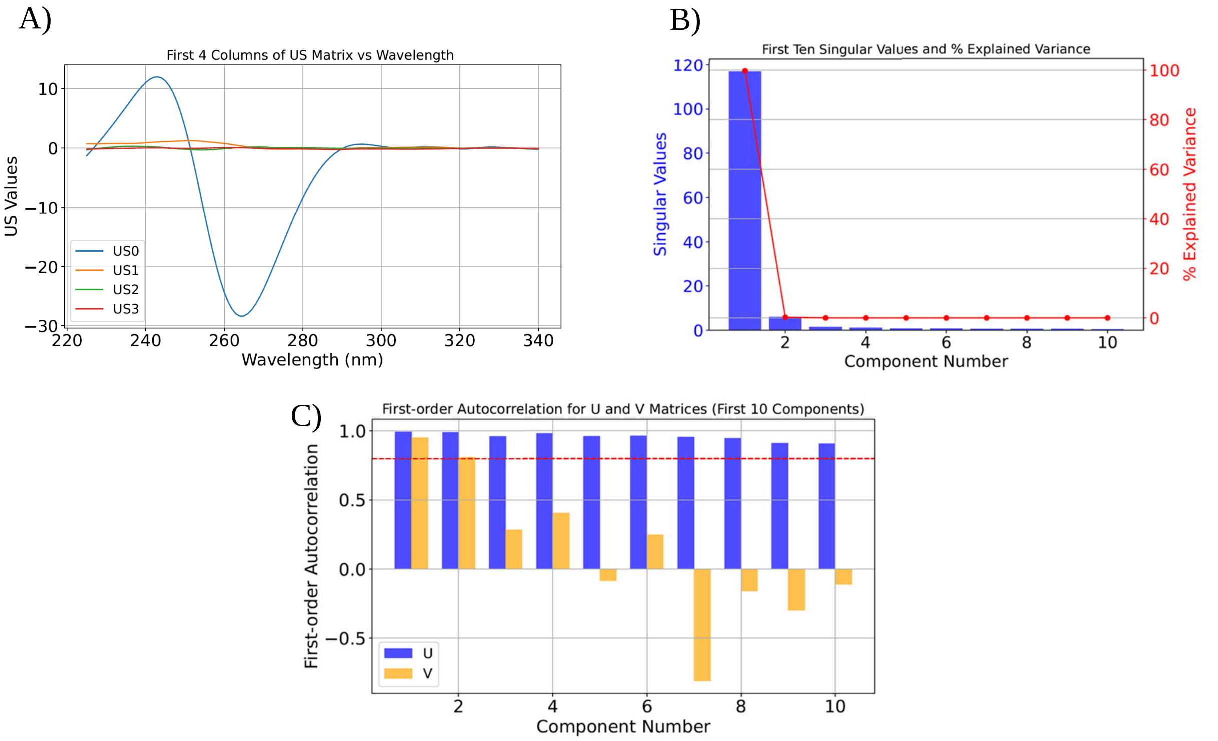

Figure SI-8. Example of singular value decomposition analysis for invTtel-Ttel in K^+ containing buffer containing two components assessed on the criteria of Gray and Chaires. A) Basis spectra of first four components weighted by their singular values. B) First 10 singular values (left-hand axis) and their contribution to the relative variance (right-hand axis). C) First-order autocorrelation of U and V columns using the suggested cutoff of ~0.8.

Instructions and Disclaimer for SVD Analysis of CD Melting Data

Disclaimer

The code provided in this Jupyter Notebook is offered in good faith. However, it is provided 'as is' without any express or implied warranties, including but not limited to the implied warranty of fitness for a particular purpose. The authors of this notebook and associated entities shall not be liable for any damages, losses, or issues that may arise from the use, misuse, or inability to use the code. Users are responsible for ensuring that the code is appropriate for their specific needs and conditions. By using this notebook, you agree to these terms.

Instructions for Using the Python Script for SVD Analysis of CD Melting Curves

1. Prepare Your Workspace

Copy the Jupyter notebook and input data files into the folder where you intend to work.

2. Ensure Required Python Packages Are Installed

Before running the notebook, ensure you have the necessary Python packages installed. You can install them using the following command in the command prompt:

pip install pandas numpy matplotlib scipy

3. Input File Format

The input data file should be in CSV format and structured as follows:

- The first column should contain the wavelengths in nanometers (nm).
- The subsequent columns should contain the CD data at various temperatures, with the temperature values (in Kelvin) as the column headers.
- Ensure that the temperature values are listed as numerical values (e.g., 288, 293, 298, etc.).

e.g.,

,288,293,298

350,0.553,0.479,0.330

349,0.127,0.292,0.451

348,0.206,0.172,0.188

4. Launch Jupyter Notebook

If using Windows, open a command prompt (cmd).
- Use the cd command to navigate to the folder containing the Jupyter notebook and data files.
- Type “jupyter notebook” or “python -m notebook” in the command window to start Jupyter Notebook.

5. Run the Notebook

In the Jupyter Notebook interface, select the code block and press the 'Run' button (the arrow icon).
- Follow the prompts as they appear.
- Note: If the notebook crashes due to a missing module, note the module name from the error

message. Open a new command prompt and install the missing module by typing pip install module_name.

6. Using Default Settings

In most cases, the default settings will be sufficient. However, you may wish to adjust the wavelengths selected for the CD intensity vs. temperature plots. The script suggests default wavelengths based on the minimum and maximum intensity values in your dataset.

7. Determine the Number of Significant Components

Refer to Gray and Chaires (ref. 33) to guide your decision on the number of significant components. Generally, this can be determined by the position of the 'elbow' in the Singular Value vs. Components plot, and by examining the autocorrelation values for the V matrix (represented by yellow bars in the histogram).
- Significant components should have autocorrelation values higher than ~0.8. If the results are marginal, inspect the subsequent plots of U and V vectors for consistency.
- Check the 'debug' output for cumulative explained variance of the singular values. Typically, two singular values correspond to two significant species (e.g., folded and unfolded). In some cases, there may be evidence of three or more significant species.

8. Recreate Plots with Significant Singular Values

Once the number of significant singular values has been determined, the script will recreate the plots of U and V vectors using only the significant singular values.
- The U vectors represent the 'basis spectra' and should resemble the CD spectrum of the corresponding species (e.g., folded, unfolded, intermediate). The V vectors approximate melting curves. Note that these may appear inverted when compared to actual spectra / melting profiles (since sign discrimination is lost in the relationship $\boldsymbol{D} = \boldsymbol{USV^T}$).

9. Fit Vectors and Identify Inflection Points

The script fits the chosen number of V vectors with a cubic spline and identifies the inflection points.
- You can adjust the smoothness of the spline to achieve the best fit (range: 0.00 for no smoothing, up to ~0.05 for a smoother curve).
- Once you are satisfied with the fit, type 'done' to complete the analysis. The script will then identify the inflection points.
- Further analysis requires fitting to a thermodynamic model with the appropriate number of components.

10. Output

The plots are saved to a PDF file, and important data is exported to an Excel workbook.
- The SVD matrix calculations were validated using the online calculator at https://comnuan.com/cmnn01004, which produced identical values for the matrices.

A)

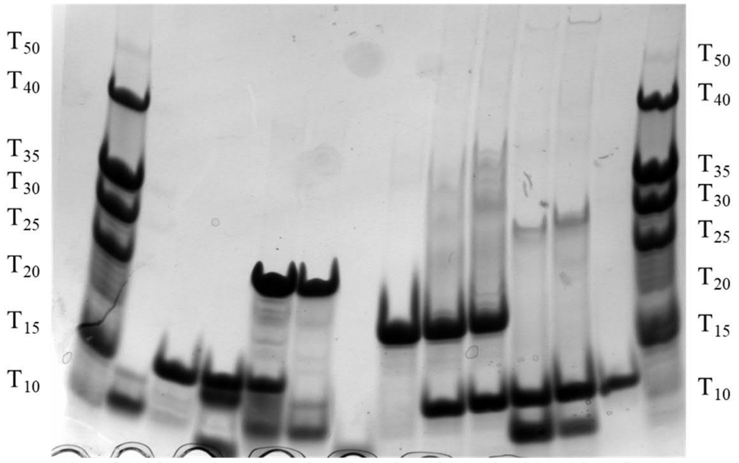

	1	2	3	4	5	6	7	8	9	10	11	12	13	14
Ttel		+	+	+	+	+								
invTtel-Ttel									+	+	+	+	+	
c-Ttel			+	+			+				+	+		
c-invTtel-Ttel					+	+		+	+	+				
Thermodynamic Product			+		+					+		+		

B)

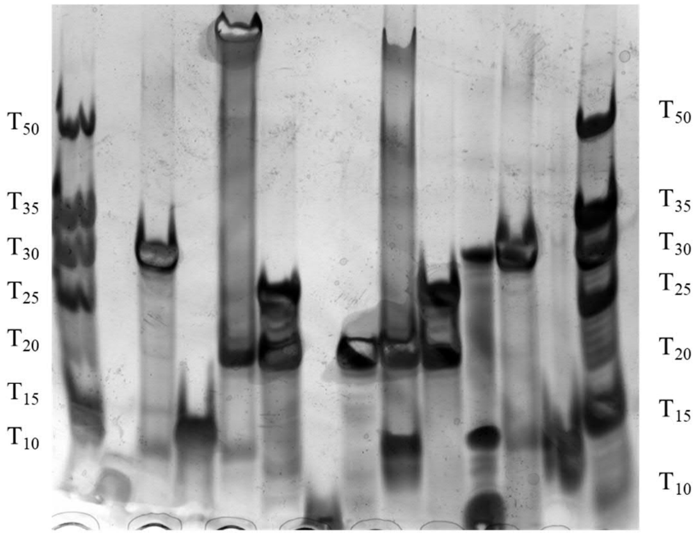

	1	2	3	4	5	6	7	8	9	10	11	12	13	14
telTA		+	+	+	+	+								
telTA-invtelTA									+	+	+	+	+	
c-telTA			+	+			+				+	+		
c-telTA-invtelTA					+	+		+	+	+				
Thermodynamic Product			+		+					+		+		

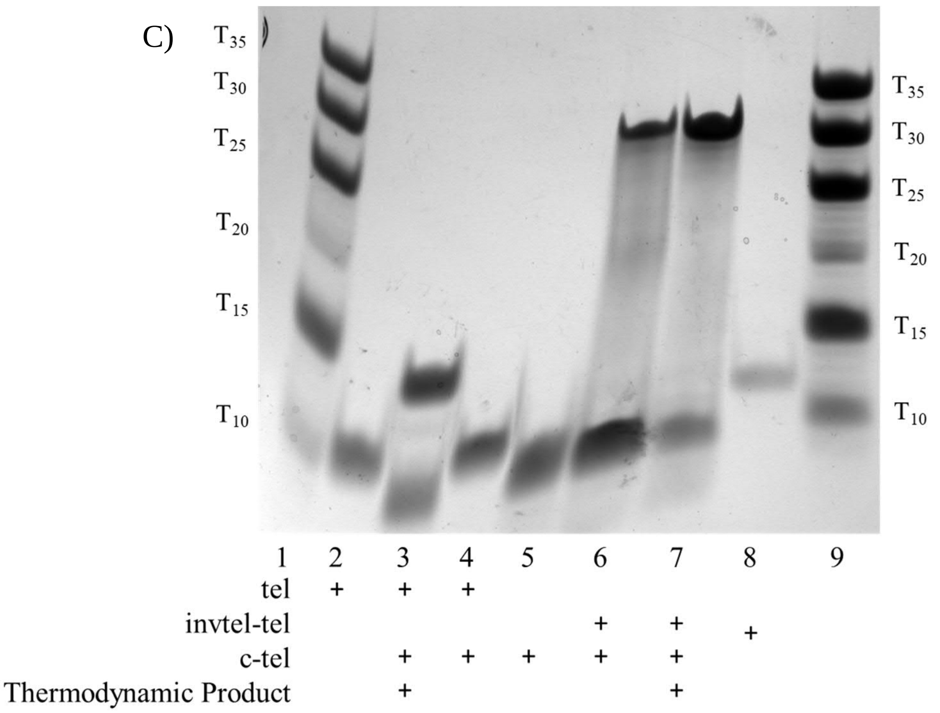

Figure SI-9. 20% Non-denaturing PAGE to observe whether formation of duplex occurs for native and inverted telomeric sequences in the presence of the complementary strand. (A) Ttel and invTtel-Ttel thermodynamic and kinetic products. (B) telTA and telTA-invtelTA. (C) tel and invtel-tel. Strand concentration: 100 µM, Buffer: TBE Buffer supplemented with 100 mM KCl. Thermodynamic product is formed by mixing G4 and complementary sequence and heating to 90°C for 5 mins, then cooling slowly to 4°C.

All short c-tel sequences (c-tel, c-Ttel, c-telTA) exhibited fast mobility on the native gel in comparison to G4, with bands present on the bottom edge of the gel (lanes 7 in A and B and lane 5 in C). These bands of intact sequences are also observed in lane 11 for kinetic products in A and B and lane 12 as thermodynamic product in A.

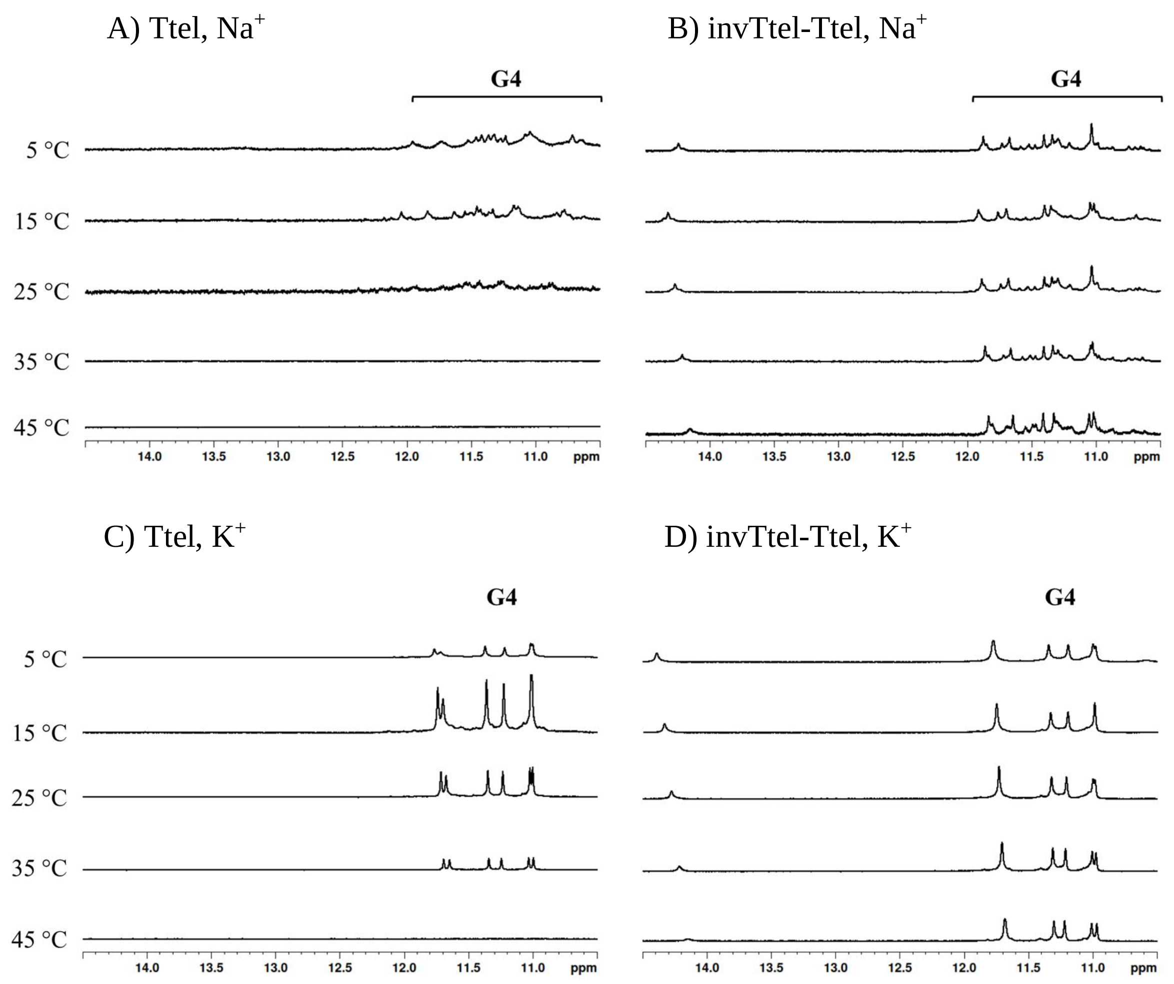

Figure SI-10. ^{1}H NMR profiles showing change in G4 structure as temperature increases, confirms results shown in SI-3 – 5. (A) Ttel, Na^+ buffer, (B) invTtel-Ttel, Na^+ buffer. Conditions: 300 µM strand concentration, buffer: 20 mM sodium phosphate, 10% D_2O, 1% TSP. (C) Ttel, K^+ buffer, (D) Ttel, K^+ buffer. Conditions: 300 µM strand concentration, buffer: 20 mM sodium phosphate, 10 mM KCl, 10% D_2O, 1% TSP, pH 7.0

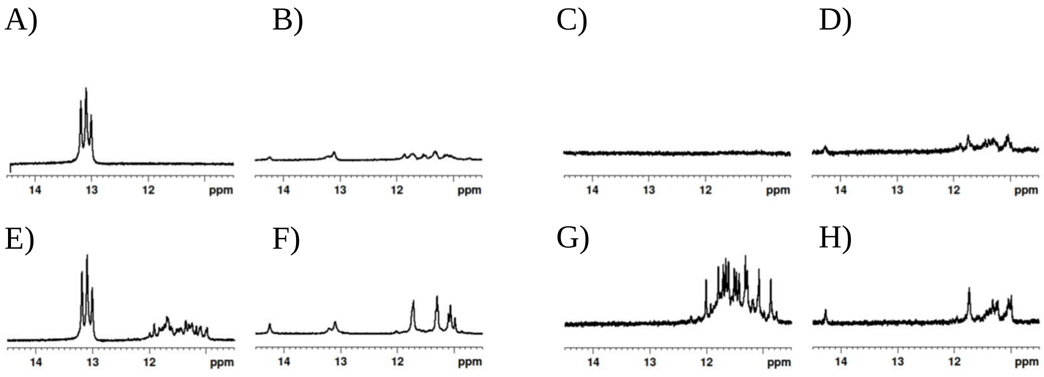

Figure SI-11. ^{1}H NMR of telomeric sequences with duplex-forming tails in different buffers. (A) Ttel-3′-Tail, Na^+, (B) invTtel-Ttel-3’-Tail, Na^+, (C) Ttel-5′-Tail, Na^+, (D) invTtel-Ttel-5′-Tail, Na^+, (E) Ttel 3′-Tail, K^+, (F) invTtel-Ttel-3′-Tail, K^+, (G) Ttel-5′-Tail, K^+, (H) invTtel-Ttel-5′-Tail, K^+. Conditions: 300 µM strand concentration, 20 mM sodium phosphate, 10% D_2O, 1% TSP; (E – H) 10 mM KCl added, pH 7.0.

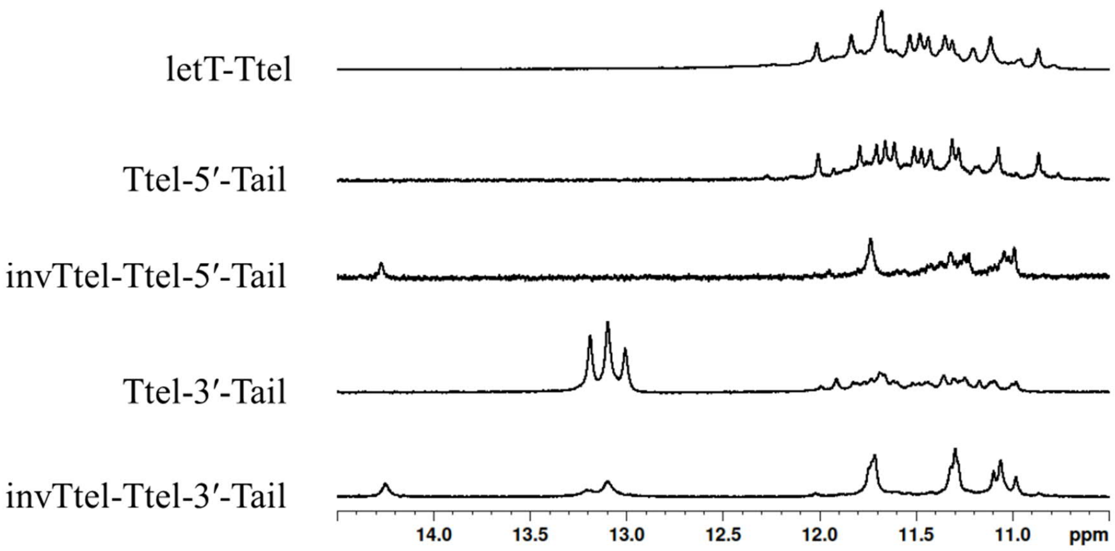

Figure SI-12. Comparison of various peaks observed in ^{1}H NMR for different G4 structures in K^{+} buffer. Peaks in native sequences correspond to each other, but inverted sequences show significantly fewer peaks. Conditions: 300 µM strand concentration, 20 mM sodium phosphate, 10 mM KCl, 10% D_2O, 1% TSP, pH 7.0.

HP1α Hinge + His-tag (underlined)

MHHHHHHDYDIPTTENLYFQGKKYKKMKEGENNKPREKSEGNKRKSSFSNSADDIKSKKKREQSNDIAR

HP1α + His-tag (underlined) – Hinge in bold
MHHHHHHDYDIPTTENLYFQGAMGSGKKTKRTADSSSSEDEEEYVVEKVLDRRMVKGQ
VEYLLKWKGFSEEHNTWEPEKNLDCPELISEFM**KKYKKMKEGENNKPREKSEGNKRKS**
SFSNSADDIKSKKKREQSNDIARGFERGLEPEKIIGATDSCGDLMFLMKWKDTDEADL
VLAKEANVKCPQIVIAFYEERLTWHAYPEDAENKEKESAKS

Figure SI-13. Amino acid sequence of HP1α with His-tag including isolated hinge region used for some BLItz experiments.
